# Why Is the Sensory Response of Organic Probes within a Polymer Film Different in Solution and in the Solid-State? Evidence and Application to the Detection of Amino Acids in Human Chronic Wounds

**DOI:** 10.3390/polym12061249

**Published:** 2020-05-29

**Authors:** Marta Guembe-García, Patricia D. Peredo-Guzmán, Victoria Santaolalla-García, Natalia Moradillo-Renuncio, Saturnino Ibeas, Aranzazu Mendía, Félix Clemente García, José Miguel García, Saúl Vallejos

**Affiliations:** 1 Departamento de Química, Facultad de Ciencias, Universidad de Burgos, Plaza de Misael Bañuelos s/n, 09001 Burgos, Spain; mguembe@ubu.es (M.G.-G.); pdperedo@ubu.es (P.D.P.-G.); sibeas@ubu.es (S.I.); amendia@ubu.es (A.M.); fegarcia@ubu.es (F.C.G.); 2 Complejo Asistencial Universitario de Burgos, 09001 Burgos, Spain; Msantaolalla@saludcastillayleon.es (V.S.-G.); nmoradillo@saludcastillayleon.es (N.M.-R.)

**Keywords:** solid-state chemosensors, sensory polymers, amino acids, chronic wounds

## Abstract

We anchored a colourimetric probe, comprising a complex containing copper (Cu(II)) and a dye, to a polymer matrix obtaining film-shaped chemosensors with induced selectivity toward glycine. This sensory material is exploited in the selectivity detection of glycine in complex mixtures of amino acids mimicking elastin, collagen and epidermis, and also in following the protease activity in a beefsteak and chronic human wounds. We use the term inducing because the probe in solution is not selective toward any amino acid and we get selectivity toward glycine using the solid-state. Overall, we found that the chemical behaviour of a chemical probe can be entirely changed by changing its chemical environment. Regarding its behaviour in solution, this change has been achieved by isolating the probe by anchoring the motifs in a polymer matrix, in an amorphous state, avoiding the interaction of one sensory motif with another. Moreover, this selectivity change can be further tuned because of the effectiveness of the transport of targets both by the physical nature of the interface of the polymer matrix/solution, where the target chemicals are dissolved, for instance, and inside the matrix where the recognition takes place. The interest in chronic human wounds is related to the fact that our methods are rapid and inexpensive, and also considering that the protease activity can correlate with the evolution of chronic wounds.

## 1. Introduction

From a chemical viewpoint, it is well known that chemicals in the solid-state exhibit completely different properties than in solution. This is because of the usual dense packaging and inter-molecule interactions in the former and the solvated state in the later, where the interaction of solvent molecules-chemical play a crucial role. This fact is well known and has been intensely exploited for different purposes, e.g., luminescence, for the preparation of cleaners, liquid crystals, catalysts, plasticisers, coatings, buffers, etc. [1]. More recently, the properties of chemicals dispersed in a polymer matrix, or chemically anchored to a polymer backbone, in the solid-state, have been exploited [2,3,4,5,6]. The substances, isolated one from another by the polymer chains or sections in the solid-sate, in an amorphous-state and interacting with the macromolecules, within a highly-motion-restricted environment, behave completely different than in solution or in conventional crystalline solid-state [2].

In this paper, we analyse the behaviour within a solid polymer matrix of a well know dye that has been previously exploited as a probe for detection of amino acids, Chromoxame Cyanine R (D), and take advantage of its different behaviour in this state than in solution to explore the ability of the new solid sensory materials, or polymer chemosensors, to detect and recognise amino acids in complex environments, such as chronic human wounds. We report on the advantage of the solid polymer chemosensors in the tuning of the properties of the chemical probes but also in the manageability of the polymer chemosensors prepared as films, that can be safely managed and stored at ambient conditions even by untrained personnel [7,8,9].

Human chronic wounds constitute a significant health societal problem. Approximately 1–2% of people worldwide suffer from a health problem that hampers the regular activity of the cutaneous wound healing process, which ultimately results in chronic wounds. Compared with cancer or with HIV, chronic wounds may not be as well known, but this condition is no less common a problem. In Spain alone, €350 million per year is devoted to the treatment of this health problem. In Europe, the total cost of chronic wounds (including nursing, medical and surgical time, hospital bed days and cost of materials) amounts to €144.000 per 100 patients. This constitutes 2–3% of the total health care budget in Europe. The USA has also performed an economic evaluation of the impact, cost, and Medicare policy implications of chronic non-healing wounds, and has concluded that the cost is nearly 32,000 million dollars [10,11,12,13]. Currently, there is no solution to this problem, although there is a medical consensus (WUWHS) [14] “that the increased activity of proteases is currently the best available marker for healing disorders when other causes have been excluded, and that the effective use of a protease analysis kit has the potential to change the treatment of wounds worldwide” [15,16]. The major players in the healing process are the proteases, these enzymes are responsible for degrading damaged proteins of the extracellular matrices (EM) in peptides and amino acids, leading to the formation of new tissues, that is, to orderly healing. The problem lies in the degradation/protein formation equilibrium, which is delicate and when it is altered by high activity of the proteases, it can lead to destroying the EM that has just been formed and other proteins such as the growth factor and its receptors giving rise to difficulties in healing. Then, knowing the level of activity of the proteases in a wound allows for the evaluation of the risk of ulceration and the bad prognosis and the probability of healing, and with this to establish palliative measures to reduce the activity of the proteases. Currently, it is complicated to evaluate the level of proteases in wounds. Regarding the analytical tests, some research studies have assessed the activity levels of the proteases in exudates used several different techniques at the laboratory level, e.g., zymography in gelatin and ELISA that uses antibodies to measure protease levels. In practice, the analytical evaluation of protease activity is not feasible for most health professionals. Concerning clinical evaluation, excessive activity of proteases may be suspected in wounds that do not heal, although clinical signs of inflammation are difficult to differentiate from those of infection.

The use of sensory materials in physiological media with medical applications has been a topical issue during the last years [17,18,19,20,21,22,23,24,25,26]. Thus, we propose herein an indirect quantification of protease activity from the quantitative detection of amino acids using sensory films. These sensory films are acrylic polymers with bidentate *N*-donor motifs (based on ethylenediamine) in the short-chain that crosslink the polymer structure, whose colourimetric sensory behaviour is based on the indicator-displacement assay (IDA) [27,28]. The ethylenediamine motifs form a complex with copper (Cu(II)) and a dye (D) giving rise to the solid sensory film. Upon immersion of the film in a water solution containing amino acids, the dye is displaced by the amino acid giving rise to a dual colourimetric signal, i.e., the colour change of the film and the colour development of the initially colourless water solution. This process is easily followed by ultraviolet/visible spectroscopy (UV/Vis) and, importantly, visually and by analysing pictures taken with conventional smartphones.

## 2. Materials and Methods

All materials and solvents were commercially available and used as received unless otherwise indicated. They included 4′-aminoacetophenone (99%, Alfa Aesar, Kandel), triethylamine (99%, VWR-Prolabo, Fonteney-sous-bois, France), methacryloyl chloride (97%, Alfa Aesar), tetrahydrofuran (THF, 100%, VWR-Prolabo), diethyl ether (99.7%, VWR-Prolabo), deuterated dimethyl sulfoxide (DMSO-d_6_, 99.8%D, VWR-Prolabo), ethane-1,2-diamine (99%, Alfa Aesar), benzene (99%, Fluka, Charlotte, NC, USA), methanol (MeOH, 100%, VWR-Prolabo), NaBH_4_ (98%, Alfa Aesar), ethyl acetate (99.9%, VWR-Prolabo), 2,2′-azobis(2-methylpropionitrile) (AIBN) (98%, Aldrich, St. Louis, MO, USA), 1-vinyl-2-pyrrolidone (VP) (99%, Acros Organics, Waltham, MA, USA), methylmethacrylate (MMA) (99%, Merck, Darmstadt, Germany), phenolphthalein (98%, Alfa Aesar), thymol blue (98%, Alfa Aesar), bromophenol blue (100%, Alfa Aesar), Eosin Y (99%, Sigma-Aldrich, St. Louis, MO, USA), fluorescein (100%, Fluka), chromoxame cyanine R (100%, Acros Organics), catechol violet (100%, Acros Organics), chorophenol red (100%, Alfa Aesar), chrome Azurol S (100%, MP), *trans*-4-hydroxy-L-proline (≥99%, Sigma-Aldrich), L-aspartic acid (98%, Alfa Aesar), L-threonine (98%, Alfa Aesar), serine (≥99%, Fluka), L-arginine (98%, Alfa Aesar), L-glutamic acid (+99%, Alfa Aesar), L-lysine (97%, Sigma-Aldrich), L-proline (99%, Alfa Aesar), L-histidine (+98%, Alfa Aesar), Glycine (99%, Alfa Aesar), L-alanine (99%, Alfa Aesar), L-cysteine (+98%, Alfa Aesar), L-valine (≥98%, Sigma-Aldrich), L-methionine (+98%, Alfa Aesar), L-isoleucine (98%, Sigma-Aldrich), L-tyrosine (+99%, Acros Organic), L-phenylalanine (98%, Alfa Aesar), N,N-dimethylacetamide (DMA, ≥99%, Sigma-Aldrich), 1-butanol (99,8%, Sigma-Aldrich), ethanol absolute (EtOH, 100%, VWR-Prolabo), 1,4-dioxane (100%, VWR-Prolabo), chloroform (99.2%, VWR-Prolabo), acetone (99%, VWR-Prolabo), acetonitrile (99.95%, VWR-Prolabo), nitromethane (95%, Sigma-Aldrich), ethylene glycol (99.9%, VWR-Prolabo), NaNO3 (≥99%, LabKem), NaH_2_PO_4_ (≥ 98%, Sigma-Aldrich), NaOH (99%, VWR-Prolabo), HCl (37%, VWR-Prolabo), sodium dodecyl sulfate (≥97%, Fluka), disodium tetraborate (99%, Sigma-Aldrich), phthaldialdehyde (97%, Merck), 2-mercaptoethanol (98+%, Alfa Aesar), CsNO_3_ (≥99%, Fluka), Mn(NO_3_)_2_ (98+%, Alfa Aesar), HAuCl_4_·3H_2_O (99.9+%, Sigma-Aldrich), K_2_Cr_2_O_7_ (≥99.5%, Sigma-Aldrich), BaCl_2_ (pure, Labkem), Zn(NO_3_)_2_·6H_2_O (98%, Aldrich), Co(NO_3_)_2_·6H_2_O (≥99%, Labkem, Barcelona, Spain), NH_4_NO_3_ (≥98%, Sigma-Aldrich), Ca(NO_3_)_2_·4H_2_O (≥99%, Sigma-Aldrich), Cr(NO_3_)_3_·9H_2_O (98.5%, Alfa Aesar), Hg(NO_3_)_2_ (98%, Alfa Aesar), RbNO_3_ (99.95%, Sigma-Aldrich), Dy(NO_3_)_3_ (99.9%, Alfa Aesar), LiCl (≥99%, Sigma-Aldrich), Cd(NO_3_)_2_ (98.5%, Alfa Aesar), Fe(NO_3_)_3_·9H_2_O (VWR-Prolabo), CeCl_3_·4H_2_O (≥99.99%, Sigma-Aldrich), ZrCl_4_ (98%, Alfa Aesar), La(NO_3_)_3_·6H_2_O (99.9%, Alfa Aesar), KNO_3_ (99+%, Sigma-Aldrich), Sm(NO_3_)_3_ (99.9%, Alfa Aesar), Mg(NO_3_)_2_·6H_2_O (≥99%, Labkem), Al(NO_3_)_2_·9H_2_O (≥98.9%, Sigma-Aldrich), AgNO_3_ (≥99.9, Sigma-Aldrich), Nd(NO_3_)_3_ (99.9%, Alfa Aesar), Pb(NO_3_)_2_ (≥ 99%, Fluka), Sr(NO_3_)_2_ (99+%, Sigma-Aldrich), Cu(NO_3_)_2_·3H_2_O (98%, Sigma-Aldrich), Ni(NO_3_)_2_·6H_2_O (98.5%, Sigma-Aldrich), sodium cyanide (>97%, Sigma-Aldrich), sodium acetate (>99%, Aldrich), lithium hydroxide (>98%, Sigma-Aldrich), sodium fluoride (≥99.9%, Sigma-Aldrich), potassium perchlorate (>99%, Sigma-Aldrich), sodium dodecyl sulphate (≥98.5%, Sigma-Aldrich), sodium nitrite (>97%, Aldrich), sodium ethoxide (95%, Sigma-Aldrich), potassium hydrogen phthalate (99.95%, Sigma-Aldrich), sodium pyrophosphate tetrabasic (>95%, Sigma-Aldrich), potassium persulfate (>99%, Sigma-Aldrich), sodium methanesulfonate (98%, Sigma-Aldrich), sodium pyrophosphate dibasic (>99%, Sigma-Aldrich), lithium trifluoromethanesulfonate (96%, Sigma-Aldrich), sodium *p*-toluenesulfonate (95%, Sigma-Aldrich), potassium bromide (>99%, Sigma-Aldrich,), potassium thiocyanate (>99%, Sigma-Aldrich), potassium oxalate monohydrate (>98.5%, Sigma-Aldrich), sodium carbonate (>99%, Sigma-Aldrich), sodium benzoate (>99.5%, Sigma-Aldrich), lithium phosphate monobasic (99%, Sigma-Aldrich,), sodium sulfate (99%, Sigma-Aldrich), sodium chloroacetate (98%, Sigma-Aldrich), sodium trifluoroacetate (>99%, Sigma-Aldrich), sodium periodate (99.78%, Sigma-Aldrich, 99.8%).

## 3. Experimental

### 3.1. Measurement Techniques

^1^H (300 MHz) and ^13^C{^1^H} (75 MHz) NMR spectra were recorded with an Inova 400 spectrometer (Varian, Palo Alto, CA, USA) operating at 399.94 MHz for ^1^H, and 100.6 MHz for ^13^C, using deuterated dimethyl sulfoxide (DMSO-*d*_6_) or deuterated chloroform (CDCl_3_) as solvents at 25 °C. Infrared spectra (FTIR) were recorded with an FT/IR-4200 FT-IR spectrometer (Jasco, Victoria, Canada) equipped with an ATR-PRO410-S single reflection accessory.

The material was thermally and mechanically characterised using thermogravimetric analysis (TGA, 10–15 mg of the sample under synthetic air and nitrogen atmosphere with a Q50 TGA analyser (TA Instruments, New Castle, DE, USA) at 10 °C·min^−1^), differential scanning calorimetry (DSC, 10–15 mg of the sample under a nitrogen atmosphere with a TA Instruments Q200 DSC analyser at 20 °C·min^−1^), and tensile properties analysis (5 × 9.44 × 0.122 mm samples using an EZ Test Compact Table-Top Universal Tester (Shimadzu, Kyoto, Japan) at 1 mm·min^−1^).

High-resolution electron-impact mass spectrometry (EI-HRMS) was carried out on a Micromass AutoSpect mass spectrometer (ionisation energy: 70 eV; mass resolving power: >10,000, Waters, Milford, MA, USA). Inductively coupled plasma mass spectrometry (ICP-MS) measurements were recorded on a 7500 ICP-MS spectrometer (Agilent, Santa Clara, CA. USA)

UV/Vis spectra were recorded using a U-3900 UV/Vis spectrophotometer (Hitachi, Tokyo, Japan). The standard experimental procedure for all measurements consisted of placing the sensory material in the bottom of a standard cuvette (1 cm side), in MeOH: pH = 7 Buffer. Next, a certain amount of amino acids is added, and the diffusion process of the dye from the sensory material to the solution was observed. The solution was always homogenised before each measurement, using a pasteur pipette.

RGB method was carried out by taking digital pictures of the sensory discs (8 mm diameter) with an iPhone 6S smartphone after immersion in aqueous media with different concentrations of amino acid. To obtain a good reproducibility of the results, as well as to avoid possible external influences in the photographs, these were taken in a dark room. The digital pictures were analysed with a generic image software to obtain the RGB parameters of the entire surface of the sensory disc. Photographs were taken three-fold for the error’s calculations, and the average of each RGB parameter was calculated. This easy and cheap method allows the quantification of amino acid in aqueous media, by only taking a photo, and we have widely used it in previous works [3,7].

Principal component analysis (PCA) was carried out using the Statgraphics Centurion XVI software (Statgraphics Technologies, The Plains, VA, USA) installed on a personal computer in a Windows 7 environment. The variable (principal component) values were standardised and accounted for >99% of the variance in all experiments

Biological samples were weighed, and for each 1 g of samples, 20 mL of pH = 7 buffer was added. Then the samples were boiled at 100 °C for 10 min to stop the hydrolysis. Finally, they were filtered hot.

### 3.2. Preparation of the Sensory Monomer

#### 3.2.1. Synthesis of N-(4-acetylphenyl)methacrylamide (1)

A mixture of 4′-aminoacetophenone (10 g, 74 mmol), triethylamine (1.6 equiv., 96.18 mmol, 9.73 g, 13.41 mL), methacryloyl chloride (1.2 equiv., 88.78 mmol, 9.28 g, 8.68 mL) and 100 mL of THF was stirred in a pressure flask at 50 °C. After four hours, the reaction mixture was filtered and the solvent was removed under reduced pressure. The solid was washed with water and then with diethyl ether. ^1^H-NMR (CDCl_3_) δ = 7.93 (d, *J* = 8.7 Hz, 2H), 7.87 (s, 1H), 7.68 (d, *J* = 8.7 Hz, 2H), 5.82 (s, 1H), 5.51 (s, 1H), 2.57 (s, 1H), 2.06(s, 1H).^13^C NMR (CDCl _3_) δ = 196.97 (C), 166.72 (C), 142.23 (C), 140.63 (C), 132.92 (CH), 129,68 (CH), 120.49 (CH), 119.16 (CH_2_), 26.41 (CH_3_), 18.67 (CH_3_). HRMS (EI) m/z [M+H]^+^ calc for [C_12_H_13_NO_2_] 204.1019; found: 204.1022 and HRMS (EI) m/z [M+Na]^+^ calc for [C_12_H_13_NO_2_] 226.0838; found: 226.0840. FT-IR (Wavenumbers, cm^−1^): ν_>N-H+_, 3350.

#### 3.2.2. Synthesis of N,N′-(((ethane-1,2-diylidenebis(azanylylidene))bis(ethane-1,1-diyl))bis(4,1-phenylene))-bis(2-methacrylamide) (2)

A mixture of ethane-1,2-diamine (0.6 g, 9.9 mmol, 0.667 mL), (1) (1.04 equiv., 20.6 mmol, 4.2 g) and 50 mL of benzene was stirred in a round bottom flask equipped with a Dean-Stark trap at 116 °C. After two hours, the reaction mixture was filtered. The filtered solid was washed with diethyl ether. ^1^H-NMR (DMSO-*d*_6_) δ = 9.89 (s, 2H), 7.89-7.65 (m, 8H), 5.83 (s, 2H), 5.54 (s, 1H), 5.51 (s, 2H), 3.79 (s, 4H), 3.34 (s, 2H), 2.23 (s, 6H) 1.96(s, 6H).^13^C-NMR (DMSO) δ = 167.26 (C), 164.39 (CH), 140.75 (CH), 135.97 (Ch), 127.28 (CH), 120,56 (CH), 120.49 (CH), 119.77 (CH_2_), 53.25 (CH), 19.16 (CH_3_), 15.43 (CH_3_). HRMS (EI) m/z [M+H]^+^ calc for [C_26_H_30_N_4_O_2_] 431.2442; found: 431.2445 and HRMS (EI) m/z [M+Na]^+^ calc for [C_26_H_30_N_4_O_2_] 453.2261; found: 453.2261. FT-IR (Wavenumbers, cm^−1^): ν_>N-H+_, 3300, ν_C-N = +_, 1660.

#### 3.2.3. Synthesis of N,N′-(((ethane-1,2-diylbis(azanediyl))bis(ethane-1,1-diyl))bis(4,1-phenylene))bis(2-methacrylamide) (3)

Compound (2) was dissolved in 25 mL of MeOH at 0 °C. Next NaBH_4_ (6 equiv., 30 mmol, 1.14 g) was added little by little. After half an hour, the reaction mixture was stirred during 2 h at room temperature. Then the reaction mixture was filtered, and the organic solvent was evaporated under vacuum yielding the impure solid. Finally, the impure solid was washed with ethyl acetate. ^1^H-NMR (DMSO) δ = 9.71 (s, 2H), 7.59 (d, *J* = 8.5 Hz, 4H), 7.22 (dd, *J* = 8.5, 1.7 Hz, 4H), 5.79 (s, 2H), 5.50 (s, 2H), 3.58 (dd, j = 8.5, 6.6 Hz, 2H), 3.33 (s, 2H), 2.35 (d, J = 2, 4H), 1.95 (s, 6H), 1.20 (d, J = 6.5 Hz, 6H). ^13^C- NMR (DMSO) δ = 167.04 (C), 141.99 (C), 140.91 (C), 137.77 (C),126.91 (CH),120.56 (CH), 120.12 (CH_2_), 57.57 (CH), 47.57 (CH_2_), 24.94 (CH_3_), 19.21 (CH_3_). HRMS (EI) m/z [M+H]^+^ calc for [C_26_H_30_N_4_O_2_] 435.2755; found: 435.2756 and HRMS (EI) m/z [M+Na]^+^ calc for [C_26_H_30_N_4_O_2_] 457.2574; found: 457.2575. FT-IR (Wavenumbers, cm^−1^): ν_>N-H+_, 3336.

**Scheme 1:**
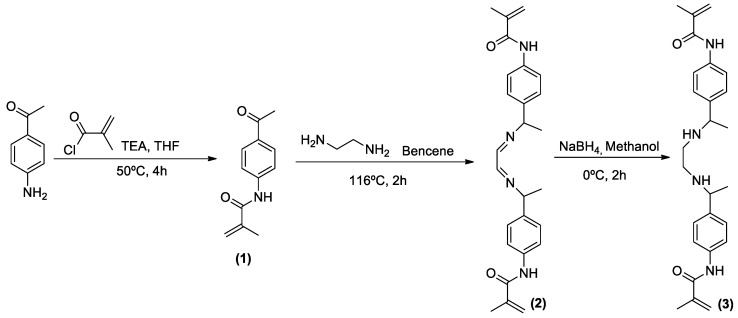
Synthetic route to the sensory monomer **3**.

Scheme 1 summarises the synthetic steps followed to prepare the sensory monomer (3). The NMR and FTIR spectra of intermediates (1) and (2) and monomer (3) are presented in the Supplementary Information (SI), Section S1, Figures S1–S3.

### 3.3. Preparation of the Sensory Film

**Scheme 2:**
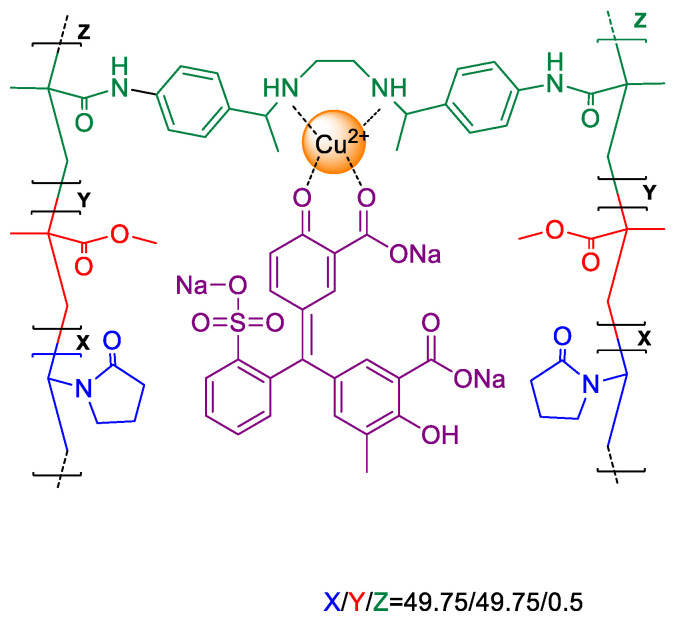
Chemical structure of sensory material **F_(3)-Cu-D_**.

The starting material **F_(3)_** was obtained by radical copolymerization of the different monomers: vinylpyrrolidone (VP) as the hydrophilic monomer, methylmethacrylate (MMA) as the hydrophobic monomer, and (3) (*N,N*′-(((ethane-1,2-diylbis(azanediyl))bis(ethane-1,1-diyl))bis(4,1-phenylene))-bis(2-methacrylamide)) as the anchorage monomer. The bulk radical polymerisation was carried out in a silanised glass mould (100 μm thick) in an oxygen-free atmosphere at 60 °C overnight. Regarding the molar ratio of the monomers, this can be adjusted for different purposes. In our case, the colorimetric response of the material toward amino acid was modulated by adjusting this molar ratio, i.e., 49.75/49.75/0.5 (VP/MMA/(3)). After the bulk radical polymerisation, the material was immersed in an aqueous solution of CuSO_4_ 0.1 M overnight. Next, the material was washed with water 5 times and the membrane **F_(3)-Cu_** was obtained. Then, the material was immersed in 100 mL of water with 1 mL of Chromoxame Cyanine R at 5 × 10^−3^ M in MeOH. Finally, the material was washed with water (3 times), acetone (twice), water:acetone (80:20) (once) and water (once) for obtaining the final sensory material **F_(3)-Cu-D_**. The chemical structure of the films used to prepare the sensory materials is depicted in Scheme 2.

### 3.4. Ethical Statement

We have performed all experiments with human subjects based on the use and ethics of the Hospital Universitario de Burgos policy for trials with humans. The ethics committee of clinical experimentation of the region of Burgos, Spain, approved this study (minute 13/2017, internal code: 2017.200) on 16 November 2017. Informed consent was obtained from all participants of the study according to Spanish Organic Law 15/199, 13 December, related to Spanish Personal Data Protection Regulation and Royal Decree-Law 1720/2007, 21 December. 

## 4. Results and Discussion

The colourimetric discrimination of enantiomeric amino acids in solution using probes of Cu(II) complexes of bidentate *N*-donor ligands was described by Anslyn et al. [27]. With this work on mind, we envisaged solid polymeric materials having bidentate *N*-donor moieties in the polymer structure to prepare complexes with Cu(II) to detect amino acids in chronic human wounds to try to correlate the response with the protease activity measured by an accepted method. Thus, our idea was to have an easy-to-manage material for the detection of amino acids, cost-effective, rapid in response.

Firstly, we prepared a crosslinked polymer with bidentate *N*-donor motifs crosslinking main polymer chains. For this purpose, a difunctional monomer (3) was synthesised following conventional procedures (Scheme 1). Then, the crosslinked film was prepared by bulk thermally induced radical polymerisation of a small quantity of the crosslinked (3) (mol 0.5%) and two commercial monomers, VP and MMA, in mol 49.75% each, to obtain a material with a proper balance of mechanical properties and gel behaviour. In this sense, VP provides hydrophilicity and MMA hydrophobicity to the prepared polymer, and the crosslinker tunes further the water swelling percentage of the materials, by physical means, giving rise to a manageable film (**F_(3)_**), even after swelling. The weight percentage of water taken up by the films upon soaking in pure water at 20 °C until reaching equilibrium (water-swelling percentage, WSP) was obtained from the weight of a dry sample film (ω_d_) and its water-swelled weight (ω_s_) using the following expression: WSP = 100 × [(ω_s_ − ω_d_)/ω_d_]. The water swelling percentage was 58.40% for **F_(3)_**, and envisaged good value for the diffusion of chemicals, such as amino acids, into the water-swollen sensory film [2,7,9].

After preparing the film, the complex of diethyamine motifs-Cu(II) within the material was easily prepared by immersing the film in a water solution of CuSO_4_ overnight (film **F_(3)-Cu_**). Then, the sensory film (**F_(3)-Cu-D_**) was prepared by immersion of the **F_(3)-Cu_** in a water solution of the dye.

**Figure 1:**
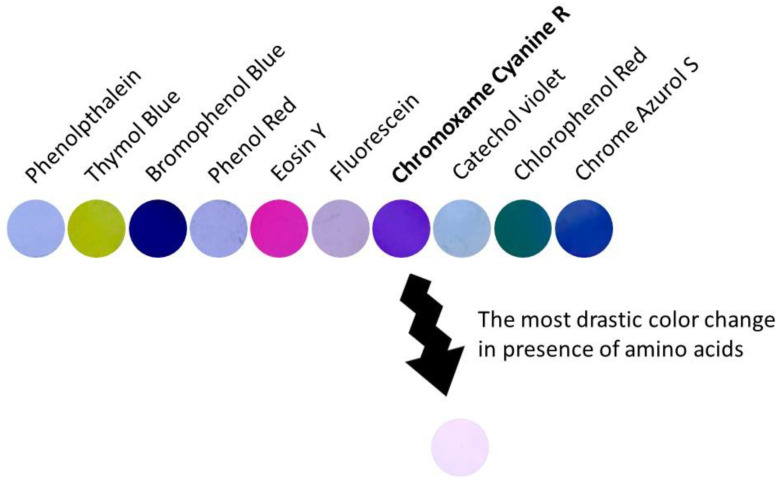
Ten discs (8 mm of diameter) of **F_(3)-Cu_** after immersion in solutions of different dyes.

The dye was chosen after screening experiments with ten different commercial dyes (Figure 1). The chosen one presented the most drastic change of colour in the presence of amino acids.

### 4.1. Mechanical and Thermal Properties of the Films

The manageability of the films is visually observed upon handling and can also be analysed by measuring the mechanical properties of the materials. Thus, Young′s moduli values for **F_(3)_**, **F_(3)-Cu_** and **F_(3)-Cu-D_** were obtained from strips of the water swelled films (Table 1). The Young′s modulus of the water swelled materials are right, ranging from 98 to 116 MPa, showing slight worsening upon diminishing the interchain interactions by the formation of the complexes between the diethylamine motifs of the polymer with Cu(II) and with Cu(II)-dye. Also, good manageability, in the broad sense, implies a reasonably good thermal behaviour, with thermal resistance well above the higher temperatures expected in the environment. Thus, the data of the thermogravimetric analysis (TGA) for the 5% (*T_5_*) and 10% (*T_10_*) weight loss under nitrogen atmosphere are higher than 300 °C (Table 1). The thermal analysis was completed with the evaluation of the glass transition temperatures (*Tg*) of the materials, that were calculated by DSC analysis at 20 °C min^−1^, obtaining values around 140 °C, higher for hybrid polymers due to the restricted motion derived from the formation of the polymer-Cu(II) and polymer-Cu(II)-dye complexes. The TGA and DSC patterns are graphically depicted in the SI–S2, Figure S4.

### 4.2. The behaviour of the Sensory Film at Different pH

The use of sensory materials usually needs specific conditions in terms of pH. For this reason, discs of **F_(3)-Cu-D_** were dipped in water solutions with pHs ranging all the conventional scale (from 1 top 14). The behaviour is depicted in SI–S3. According to the results, the sensory material can be used in a broad pH range (5 to 10). Thus, a biological pH was used for this study, pH = 7.

### 4.3. Interference Study

The discs of sensory film **F_(3)-Cu-D_** were immersed for 24 h in a buffered solution of water/MeOH with a broad set of cations and anions (see SI–S4). The blue colour of the film changed its colour only in the presence of three cations, Au(III), Fe(III) and Nd(III). Among these cations, only Fe(III) can be considered an interferent because it is frequently present in biological samples. For this reason, a thorough study was carried out with this cation, at a concentration of 2.7 × 10^−5^ M (1.5 ppm), the maximum content of iron in the blood. Moreover, as biological samples are diluted 20 times their weight in the proper mixture of solvents, previous measurement, the maximum concentration of iron in the derived solution is 1.4 × 10^−6^ M (75 ppb). This concentration caused no interference at any measuring time, and accordingly, the sensory material can be used safely without the interference of any cation or anion. 

### 4.4. Why Films Are Not Only a Support but Deeply Influence the Performance of the Sensory Probe?

It is usually found in the bibliography that solid polymers having sensory motifs chemically anchored to the polymer backbone behave in a different way than the sensory chemicals in solution beyond the lack of migration of the sensory motifs to the solution. It is stated that these systems mimic enzymes in water solutions that have a broad hydrophilic body that maintains the protein hydrated (tertiary structure) and small hydrophobic active sites where selective reactions take place without the competition of water [2,3,5,29]. Herewith we demonstrate the influence of the polymer matrix in outperforming the performance of conventional probes by two means: analysing the interaction of the probe or sensory motifs in solution and chemically anchored to a polymer in the solid-state and analysing the influence of the diffusion of species in solution into the swelled film.

### 4.5. Analysis of the Interaction of the Dye with Solvents both in Solution and in the Amorphous and Solid-State (within the Solvent Swelled Film)

As has been previously mentioned, chemicals in the solid-state exhibit different properties than in solution, and this fact can be exploited for other purposes. Thus, chemicals dispersed in a polymer matrix, or chemically anchored to a polymer backbone, in the amorphous state, and isolated one from another by polymers chains, or sections, with high restriction in movement, exhibit different properties because their chemical environment is different. For instance, highly thermally labile chemical groups, that cannot be isolated in conventional organic chemistry, such as diazonium salts, can be used without care at room temperature, for weeks, and exploited as sensors in sensing harmful chemicals in water media, when anchored to polymer backbones [2]. It is as if the polymer chains generate a protective environment for the labile group, and generalising, the polymer chains deeply influence the interactions that the chemicals within the polymer matrix can establish with others. 

For getting the insight of the environment of a chemical in solution and the isolated amorphous state within a polymer matrix, we have analysed the interaction of solvents with the dye D by UV/Vis spectroscopy.

The influence of the solvent on the absorption spectra in the visible/ultraviolet range is due to the differences in solvation between the fundamental and excited state of the chemical (solute) [1]. These appear when there is an appreciable difference in the distribution of the population between the two states, often accompanied by a significant change in the dipole moments. The effect of the solvent is called solvatochromism and is described in terms of the displacement of the position of the peak of lower energy, with a greater wavelength, in the absorption spectrum. This can be to shorter wavelengths, hypsochromic (blue shift, negative solvatochromism), or to longer wavelengths, bathochromic (redshift, positive solvatochromism). The first effect takes place when the fundamental state is more dipolar than the excited state, while the opposite occurs when the excited state is more dipolar. These changes refer to the variation of energy between the states [30].

Non-polar solutes, in the presence of both polar and non-polar solvents, mainly experience dispersive forces, the effect thereof being very small and bathochromic, which increases with the polarity of the solvent. For polar solutes in non-polar solvents, the two types of displacements increase with solvent polarizability, depending on the dipole moment of the fundamental or excited state. When both are polar, the situation is more complex, since there is a rearrangement of the solvent around the solute in both the fundamental and the excited state, so that both the polarizability and polarity of the solvent and the induced polarisation of the solute influence the displacements.

The absorption spectra are generally due to transitions *n*→π*, *π*→π* and electronic charge transfer. The most popular solvatochromic model currently in use is the Taft-Kamlet method [31,32,33,34]. In this model, multiple parameters are implemented to characterise different solvent-solute interactions, in the form of Equation (1):

(1) $$\upsilon =\upsilon_{o}+s\left(\pi^{*}+d\delta \right)+a\alpha +b\beta$$

where *υ* is the energy of the transition, the inverse of the wavelength, *υ*_o_ is the energy of the transition in the absence of solvent, π^*^,α y *β* describe the polarity of the solvent, acidity or ability to donate a proton to a hydrogen bond (HBD) and the basicity or ability to accept a proton from a hydrogen bond (HBA) respectively (SI–S5, Table S9) [31,33]. *δ* is a correction term introduced due to the different polarizability of aromatic and polychlorinated solvents concerning aliphatic and non-polychlorinated solvents, 0 being for aliphatic solvents not substituted with chlorine, 0.5 for polychlorinated aliphatic and 1 for aromatic solvents. If the solvent causes positive solvatochromism, this correction term is not necessary, and the coefficient d is zero. The coefficients *s*, *d*, *a* and *b* quantify the contributions of these properties. When working with-non-chlorinated or non-aromatic solvents and if *a* and *b* are very small, the equation can be simplified to Equation (2), and s can be easily obtained from the slope of the linear fitting of π^*^ and *υ* data (SI–S5, Figure S10).

(2) $$\upsilon =\upsilon_{o}+s\pi^{*}$$

Following the results obtained, it follows that the predominant effect is the polarity of the solvent on the dye, stabilising the fundamental state more than the excited one, producing displacements at shorter wavelengths as the polarity of the solvent increases. Meaningful dipole-dipole interaction is observed in the case of dyes in solution, with a value of *s* = 3.22. However, when the dye is in the film, having Cu(II) or not, the value of *s* is very small, around 0.6. This data indicates that the environmental surroundings of dye motifs prevent or hinders the dipole-dipole interactions between the dye motifs and the solvent swelling the film. Somehow it can be said that the structure of the film protects the dye.

### 4.6. Diffusion of Species in Solution into the Swelled Film

In the literature, we find numerous works dealing with adsorption of substances, generally pollutants, in different substrates with variable particle size [35,36,37,38,39]. In this work, we may consider the sensor as an adsorbent and the target amino acids as adsorbant. In our case, the adsorbent is not a particle of a certain size, but a 100 μm thick membrane.

In the adsorption processes in solution, several stages of transport take place in series: (a) external transport of the adsorbate moving within the solution to nearby of the nearness of the sensory film. This is a quick process; (b) diffusion of the adsorbate towards the surface of the sensory film, or external mass transfer; (c) intraparticle diffusion; (d) adsorption itself on the sensory motifs.

There are two approaches in mathematical modelling for the kinetic study of adsorption: surface reaction model (SRM), where the mass transfer is assumed to be rapid and the adsorption reaction (step d) is the stage that limits speed; and model of mass transfer reaction (MTM), where the mass transfer is the slow stage while the adsorption reaction is rapid. In this late-model there are two possibilities, single resistance model (intraparticle or external diffusion) or dual resistance model, i.e., both intraparticle and external diffusion playing an important role where steps (b) and (c) control the adsorption speed.

All these models were analysed in-depth, and the results are shown in the SI, Section S6. The model that gave us better results was the dual resistance model that considers both intraparticle and external diffusion in the analysis of the data, where relevant data were obtained following the methodology described by Crank [40] from Equations (3)–(5), where *q_t_* is the milligrams of adsorbate per gram of adsorbent at a time t, *q_e_* are equilibrium sorbate concentration in sorbent (mg/g), *B_i_* is Biot number, i.e., ratio of external film diffusivity to intraparticle diffusivity, *D_s_* diffusion coefficient of sorbate within the sorbent (cm^2^/min) and *k_f_* are external or film mass transfer coefficient, (cm/min):

(3) $$\frac{q_{t}}{q_{e}}=1-\overset{\infty }{\underset{n=1}{\sum }}\frac{2B_{i}^{2}exp\left(-\beta_{n}^{2}D_{s}t/l^{2}\right)}{\beta_{n}^{2}\left(\beta_{n}^{2}+B_{i}^{2}+B_{i}\right)}$$

(4) $$\beta_{n}tan\beta_{n}=B_{i}$$

(5) $$B_{i}=\frac{k_{f}l}{D_{s}}$$

**Figure 2:**
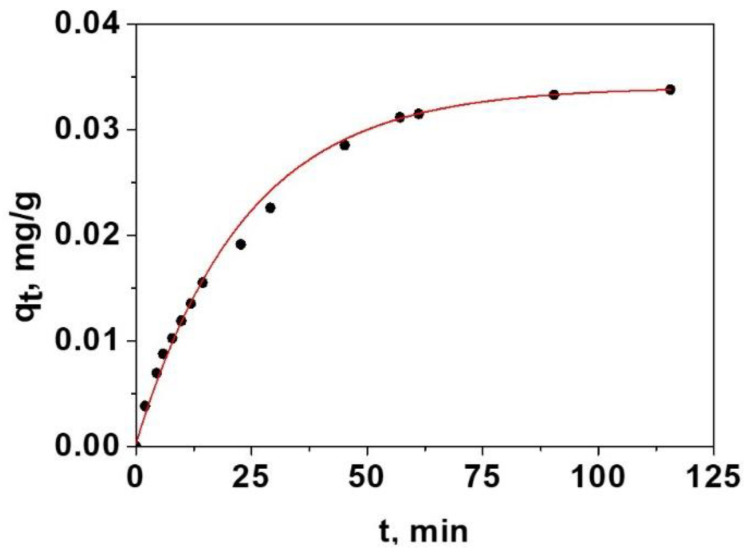
Non-linear fitting of the data of adsorption of glycine in the sensory film using the Crank model of dual resistance Equations (3)–(5).

With Equations (3)–(5) the parameters *D_s_* and *k_f_* were optimised by a non-linear fitting by least squares. This model, for spherical particles, has been used by other researchers [41]. In this way, following by UV-V is technique the liberation of D in the presence of amino acid is possible to calculate the adsorbed mg of amino acid, i.e., the adsorption curve. Figure 2 shows the results obtained for one of the amino acids, glycine.

Once estimated the external and internal diffusion coefficients for a given adsorption system, the speed limitation step can be determined in terms of the number of Biot, *Bi*, which relates the external mass transfer resistance to the resistance of internal mass transfer, Equation (5). 

When the *Bi* >> 1, the adsorption process mainly controls by intraparticle diffusion, and if *Bi* << 1, it is the external diffusion that primarily controls the speed [42,43,44]. Once the values of *Bi* have been checked, Table 2, the stage that mainly controls this adsorption process is diffusion over the boundary layer or the transport of external mass.

In our sensory system, this fact means that the solid sensory film exerts, because of the diffusion over the boundary layer, restrictions in the transport of chemicals favouring the diffusion of one chemical species against others, thus introducing a physical selectivity that does not apply for probes in solution. In our case, this selectivity favours glycine (Table 2). Furthermore, one the glycine is inside the solid sensory films, its interaction with the sensory motifs is facilitated by the limited interaction of these sites with the solvent, in our case water. 

### 4.7. Analysis of the Complex Formation between the Polymer, Cu(II) and Dye

**Figure 3:**
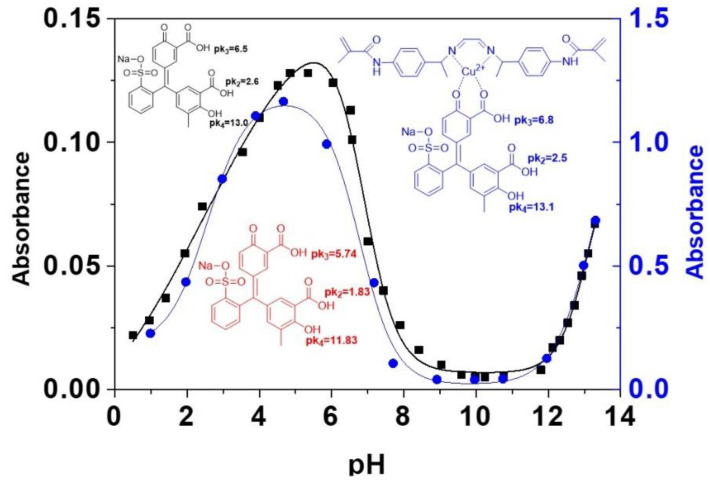
Red: pKa values for D according to the literature in aqueous media. **Black**: pH study and obtained pKa values for D in methanol:pH 7 buffer solution (1:1) media. **Blue**: pH study and obtained pKa values for the complex {(3)-Cu(II)}nDm (n:1, m:1) in methanol:pH 7 buffer solution (1:1) media.

For getting an insight of the complex formation of the polymer with Cu(II) and the dye, we have analysed by UV/Vis the complex formation in a solution of the monomer (3) with Cu(II) and the dye D. Thus, to a DMA solution of D increasing concentration of a solution of (3) and Cu(II) in a molar ratio of 1:1. The UV/Vis spectra (SI–S7, Figure S17) showed one isosbestic point at about 350 nm (confirming the interaction between D and {(3)-Cu(II)} at 1:1 stoichiometry), allowing for the analysis of three processes corresponding to the formation complexes {(3)-Cu(II)}_n_D_m_ with stoichiometries n:m of 1:1, 2:1, and 3:1. The later was also estimated with a Job′s plot (SI–S7, Figure S18). However, at high ratios of dye to (3)-Cu(II), as it is in the preparation of the sensory film, the expected stoichiometry is 1:1. The study of the behaviour of the dye D and the complex {(3)-Cu(II)}_n_D_m_ (n:1, m:1) at different pH by UV/Vis allowed for the determination of the pK_a_s of the acid protons. Figure 3 depicts the pK_a_s, both reported in previously [45] (aqueous media) and calculated by us (methanol: pH = 7 buffer solution, 1:1). pK_1_ values given in the literature are outside of the pH scale; thus, we have determined only pK_2_, pK_3_ and pK_4_.

### 4.8. Colourimetric Sensing of Amino Acids

The immersion of discs of **F_(3)-Cu-D_** in an aqueous/methanol solution buffered at physiological pH gave rise to the discolouration of the initially blue films and the colouration initially colourless solution. The interaction of the amino acids with the **F_(3)-Cu-D_** lead to the initial material **F_(3)_**, with the concomitant displacement of the dye and the formed new colourless complex Cu-(amino acid) [46,47,48,49,50,51,52,53,54,55,56,57], initially to the swelled film and finally to the solution.

Both the discolouration of the films (image analysis of pictures taken of film) and the colour evolution of the solution (UV/Vis technique) can be used to detect the presence of amino acids in solution, and to measure their concentration upon previously construction of titration curves using known concentrations of amino acids.

Starting with the UV/Vis analysis based on the colour evolution of the solution, and to test the viability of the sensory system in complex environments, solutions of 7, 4 and 18 amino acids mimicking collagen, elastin and epidermis were initially prepared (see SI–S8, Tables S19–S21) [58,59,60]. Then, discs of **F_(3)-Cu-D_** were immersed in water/methanol buffered at pH = 7 in different vials and they were spiked with these solutions to give different apparent concentrations (sum of the concentration of amino acids in each solution). The absorbance along time for all the vials allowed for the calculation of the kinetic rate constants inside the sensory film [61], which are shown for collagen, elastin and epidermis in the SI–S9, Tables S22–S24. Since the absorbance value is proportional to the concentration of adsorbed glycine, the ratio between A and time is a point measure of rate. In the first measures of the reaction, the graphical representation of A/t against t is a straight line, and its origin is the value of the initial rate. The time for kinetic measurements ranged 1–5 min.

**Figure 4:**
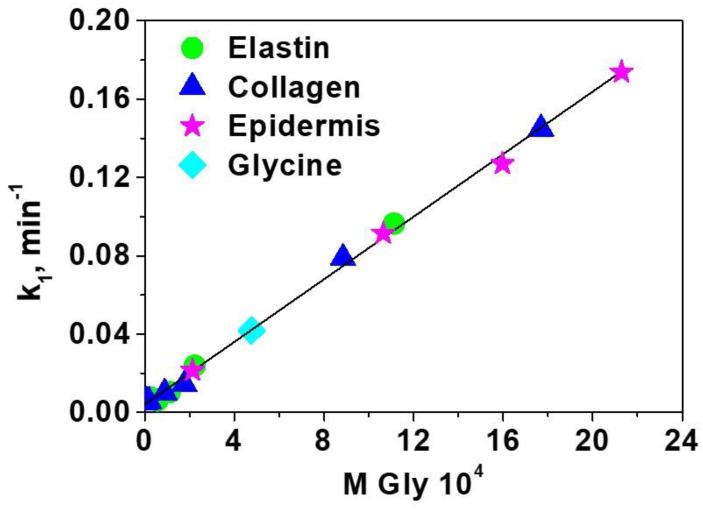
Kinetic rate constants of the kinetic of displacement of the dye by amino acids in the solid sensory **F_(3)-Cu-D_**. The rate constants were calculated using the UV/Vis absorption data obtained from the solution where the sensory discs were immersed.

Thinking in the idea that the sensory film may be selective to one amino acid, as commented before, specifically glycine, the data of rate these rate constants were plotted against the concentration of glycine, giving a straight line, as shown in Figure 4. Moreover, the data for the three complex systems is fully comparable with that of one with only glycine. This relevant information means that we have selective sensory material toward glycine, even though the sensory motif in solution responds equally to all amino acids. The good fitting of the different systems (collagen, elastin and epidermis) to a pseudo-first-order model (as glycine), is due to the high molar proportion of glycine in this system (37.15%, 46.71% and 44.71% respectively). The limits of detection and quantification of glycine are 1.9 × 10^−5^ and 5.7 × 10^−5^ M, respectively.

**Figure 5:**
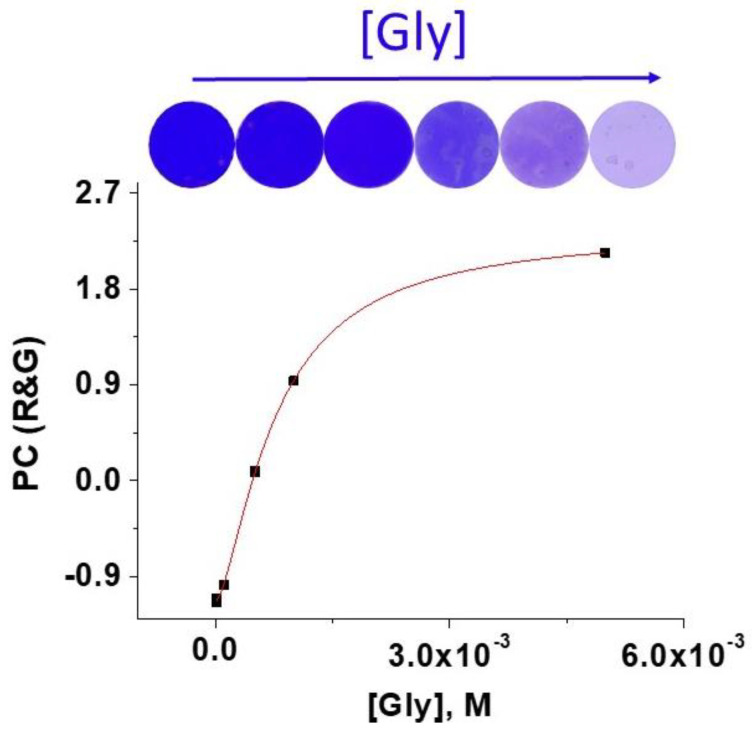
Titration of with Gly with **F_(3)-Cu-D_** by RGB method. The top of the figure shows the photographs of **F_(3)-Cu-D_** discs after immersion for 60 min in 0.63 mL of MeOH: pH 7 buffer solution (1:1) for concentrations of glycine ranging from 1 × 10^−6^ M to 5 × 10^−3^ M.

To achieve lower detection times, initial rates at 2 and 5 min were also calculated and correlated with amino acid concentration. Methodology and results are shown in the SI, Section S10. Once analysed the selective detection of glycine in complex mixtures of amino acids by studying by UV/Vis the colouring of the solution in which a sensory film was immersed, the colour decreases of the sensory films upon contacting with glycine was studied. This time, the colour variation was analysed by studying the digital colour definition of a picture taken to the discs. The digital colour was defined by three variables according to the RGB model (Red, Green and Blue), having these variables values between 0 and 255. To have the meaning of the two relevant variables (R and G) in only one, they were reduced to a single variable call PC (principal component) by the principal component analysis (PCA) [8]. The titration curve is depicted in Figure 5 for solutions containing different concentration of glycine in which the sensory discs were immersed for 60 min (the RGB data are shown in SI–S11, Figure S28, along with relevant PCA data). The limit of detection and quantification of glycine was 1.6 × 10^−4^ and 4.7 × 10^−4^ M, respectively. Though these values are one order of magnitude higher than those obtained with the previous method that uses UV/Vis spectroscopy, the lack of need of laboratory equipment using only a digital camera as analysing technique makes this method especially valuable. The analysis was also carried out with epidermis, at immersion times of 1 and 5 min, showing that quick measures can be carried out (SI–S11, Figures S29 and S30).

The comparison of published detection methods for amino acids is shown in Table 3. These methods have advantages and disadvantages, showing our proposal advantages related to the inexpensive methodology, visual detection and low response time.

### 4.9. Proof of Concept. Sensing Amino Acids from a Beefsteak and A Human Chronic Wound

**Figure 6:**
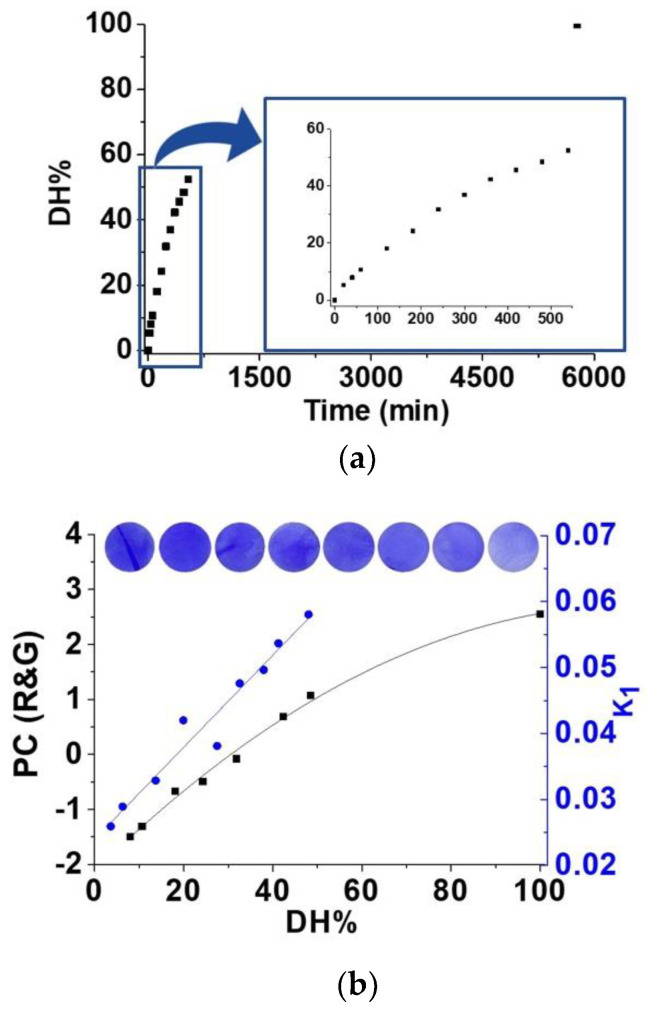
(**a**) Degree of hydrolysis of a beefsteak obtained by UV/Vis using the Nielsen et al. procedure (DH% = [(A_sample_ − A_blank_)/(A_100%_ − A_blank_)] × 100; A = absorbance) vs. time [62]. *Experimental conditions*: 12.5 g of beefsteak and 62.5 mg of papain were made up to 250 mL with pH 7 buffer solution. The mixture was stirred at 50 °C and aliquots of 5 mL were taken at different times. Additional information in SI-S12. (**b**) *Blackline:* Correlation of the RGB method with DH%. PC (R&G) parameter was obtained by multivariable analysis of parameter R & G from digital images of the discs of **F_(3)-Cu-D_**, after immersion for 60 min in the filtered aliquots from the hydrolysis reaction. *Blueline*: Correlation of the kinetic rate constants of the kinetic of displacement of the dye by amino acids in the solid sensory **F_(3)-Cu-D_**. The rate constants were calculated using the UV/Vis absorption data obtained from the solution where the sensory discs were immersed.

The first proof of concept carried out was the detection of amino acids coming from the action of the proteinase papain (papaya proteinase I) on a beefsteak bought in the local market (SI–S12, Figure S31). The degree of hydrolysis (DH%) caused by papain is usually followed by UV/Vis using the procedure described by Nielsen et al. [62] (Figure 6a). We have also analysed the digital colour of our sensory discs dipped in partially hydrolysed beef steak samples and correlated with the previously calculated DH%, showing a good correlation and confirming the viability of our analytical procedure for providing information about the content of amino acids of a solution (Figure 6b, black line).

Similarly, both the kinetic rate constants of the kinetic of displacement of the dye by amino acids of the partially hydrolysed beef steak in the solid sensory **F_(3)-Cu-D_** (Figure 6b, blue line) and the initial rates at 1 and 5 min calculations (SI–S12, Figure S32) achieved using the UV/Vis absorption data obtained from the solution where the sensory discs were immersed, showed a good correlation with the DH% obtained with the method of Nielsen et al. [62].

The second proof of concept was carried out with different types of samples (swab, wound bed, edge, capsular tissue and bone) of a chronic wound of the same patient. After, treating samples according to the procedure described in the SI–S13, the supernatant has the amino acids of the samples derived from the action of the different proteases over the proteins. The amino acid content of samples was characterised by the reference method, as previously explained. Then sensory discs were immersed at different times in the biological samples, and the colour of the films was analysed by the previously described procedure from the pictures taken to them. No results were obtained, probably because of the low concentration of the amino acids in these solutions. Also, after the immersion of each disc in the biological samples, they were evaluated by UV/Vis along time, and the kinetic rate constants evaluated. This time the method was sensitive enough to detect the amino acids, and this is so because the limits of detection and quantification of this method are one order of magnitude lower than those obtained analysing the pictures taken to the film. Accordingly, the rate constants were proportional to the absorbance at 330 nm. This means that the sensory material can probably be used to follow the evolution of the chronic wounds and may be of help to assist doctors treating chronic wounds, especially because the analysis is rapid (less than 60 min after taken the samples) and costless (each disc can be fabricated at a lab-scale for less than 0.1 euros).

### 4.10. Reusability of the Sensory Material

The sensory discs can be used to detect amino acids, washed and used again for at least 6, as shown in the SI–S14, Figure S34, without an apparent loss of performance.

## 5. Conclusions

The presented research deals with the differences in the chemical behaviour of chemical probes in solution and the solid-state and the way of exploiting these differences in the field of polymeric chemical sensors. Thus, we previously looked for a sensory complex, previously exploited in solution for the colourimetric detection of amino acids, and then we anchored this sensory motif to a polymer backbone in the solid-state that gives rise to a film-shaped sensory material for discussing the selectivity and sensitivity of the solid materials compared with that of the probe in solution. Therefore, we report herein on the influence of the chemical environment of the amorphous and isolated sensory motifs within the solid polymer matrix, and also on the influence of this matrix in developing selectivity due to restrictions in the transport of target species into the material where the sensory motifs can interact with the target. More precisely, we have found that the sensory material, as a whole, is selective to glycine among several amino acids, whereas the probe in solution it is not, and we have applied it to the detection of amino acids in human chronic wounds, as a first step in the correlation of chronic wound evolution with the protease activity within the wound. Furthermore, the solid-state is promising for exploiting organic or inorganic motifs with different and tuned properties related with the lack of interaction between these motifs that are in the amorphous state, the interaction with the polymer chains that can be tuned in terms of hydrophilic or hydrophobic behaviour, and the interface between the polymer matrix and the liquid or gaseous media.

## Figures and Tables

**Table 1:** Mechanical (Young modulus, MPa) and thermal behaviour (degradation temperatures: 5% (*T_5_*) and 10% (*T_10_*) weight loss; thermal transition: glass transition (*Tg*)) of films.

Polymers	Mechanical Properties of Water Swelled Films, Young Modulus (MPa)	Thermal Properties, in N_2_		
		Thermal Resistance		Thermal Transition
		*T*_5_ (°C)	*T*_10_ (°C)	*Tg* (°C)
F_(3)_	116	355	372	137
F_(3)-Cu_	108	355	374	144
F_(3)-Cu-D_	98	347	366	140

**Table 2:** Parameters obtained by non-linear least-squares adjustment of Equation (3).

Amino Acid	k_f_ 10^4^, cm/min	D_s_ 10^4^, cm^2^/min	B_i_	R
Arginine	0.31 ± 0.07	0.009 ± 0.001	0.17 ± 0.06	0.9975
Aspartic Acid	0.47 ± 0.02	0.021 ± 0.006	0.11 ± 0.04	0.9977
Phenylalanine	0.57 ± 0.09	0.024 ± 0.004	0.12 ± 0.04	0.9989
Glutamic Acid	0.89 ± 0.02	0.038 ± 0.007	0.12 ± 0.02	0.9989
Hydroxyproline	1.18 ± 0.04	0.041 ± 0.003	0.14 ± 0.02	0.9977
Proline	1.41 ± 0.05	0.049 ± 0.003	0.14 ± 0.01	0.9968
Alanine	1.75 ± 0.05	0.067 ± 0.003	0.13 ± 0.01	0.9981
Valine	2.00 ± 0.1	0.082 ± 0.004	0.12 ± 0.01	0.9975
Glycine	2.20 ± 0.1	0.092 ± 0.003	0.12 ± 0.01	0.9973

**Table 3:** Comparative table of different amino acid analytical methods.

Method	Detection Method	Low Cost	Response Time	Naked Eye Detection	Limit of Detection	Reference
Reference method	UV-vis	no	15 min	no	-	[62]
Screening method	Chromatography	yes	5 min	yes	qualitative method	[63]
hyperpolarised ^13^C-^1^H-2D-NMR	NMR	no	4 h	no	-	[64]
Trichophyton mentagrophytes var. erinacei	UV-vis	no	2 w	no	-	[65]
CE-LIF	Electrophoresis and Fluorimetry	no	4 h	no	25–50 nM	[66]
HPLC	HPLC	no	3 h	no	50–60 fmol	[67]
HPLC-CLND	HPLC (CLND)	no	3 h	no	0.0025–0.0075 mM	[68]
High Voltage Electrophoresis	Electrophoresis	no	22 h	no	-	[69]
Chromatography	Chromatography	no	3 h	no	0.5–10 µg	[70]
Screening method	Chromatography	no	24 h	no	1.5–7.5 w%	[71]
RGB	Digital pictures (RGB parameters defining the digital colours)	Yes	5–60 min	yes	1.58 × 10^−4^ M	This work
Kinetics	UV-vis	Yes	5–300 min	yes	1.89 × 10^−6^ M	This work
Initial rate	UV-vis	yes	1–5 min	yes	1.2 × 1 0^−4^ M	This work

## Supplementary Materials

The following are available online at https://www.mdpi.com/2073-4360/12/6/1249/s1, FTIR, UV/Vis and CIE diagrams, SEM images, ^1^H-NMR and ^13^C-NMR spectra of model molecules, synthesised organometallic polymers and films obtained.
