# Supplementary material for: Why Is the Sensory Response of Organic Probes within a Polymer Film Different in Solution and in the Solid-State? Evidence and Application to the Detection of Amino Acids in Human Chronic Wounds
Source: Polymers (Basel). 2020 May 29;12(6):1249. doi: 10.3390/polym12061249 (PMC7362178; doi:10.3390/polym12061249)
Supplement: Supplementary file 1 [file polymers-12-01249-s001.pdf]

Electronic Supplementary Information (ESI)

Why the sensory response of organic probes is different in solution and in the solid-state within a polymer film? Evidence and application to the detection of amino acids in human chronic wounds

Marta Guembe-García,[a] Patricia D. Peredo-Guzmán,[a] Victoria Santaolalla-García,[b] Natalia Moradillo-Renuncio,[b] Saturnino Ibeas,[a] Aranzazu Mendía, Félix C. García,[a] José Miguel García,* Saúl Vallejos[a,*]

[a] Departamento de Química, Facultad de Ciencias, Universidad de Burgos, Plaza de Misael Bañuelos s/n, 09001 Burgos, Spain. Email: jmiguel@ubu.es
[b] Complejo Asistencial Universitario de Burgos, Burgos, Spain

Table of contents:

S1 Characterization of the sensory monomer........ 2

S2 Thermal characterization of Materials........ 5

S3 The behaviour of the sensory film at different pH........ 6

S4 Interference study........ 7

S5 Interaction of the dye with solvents........ 8

S6. Diffusion of species in solution into the swelled film........ 9

S7. Complex formation between the polymer, Cu(II) and dye........ 15

S8 Preparation of the solutions mimicking collagen, elastin, and epidermis........ 17

S9 Kinetic constants for collagen, elastin and epidermis and different Σ molarities (*Σ M*)... 18

S10 Calculation of initial rates at 1 and 5 min........ 19

S11 RGB method........ 21

S12 Proof of concept 1. Hydrolysis of a sample of food matrix (beef, loin cut)........ 24

S13 Proof of concept 2. Biological samples from chronic wound........ 26

S14 Reversibility of the material........ 27

S1. Characterization of the sensory monomer

- ***Synthesis of N-(4-acetylphenyl)methacrylamide (1).***

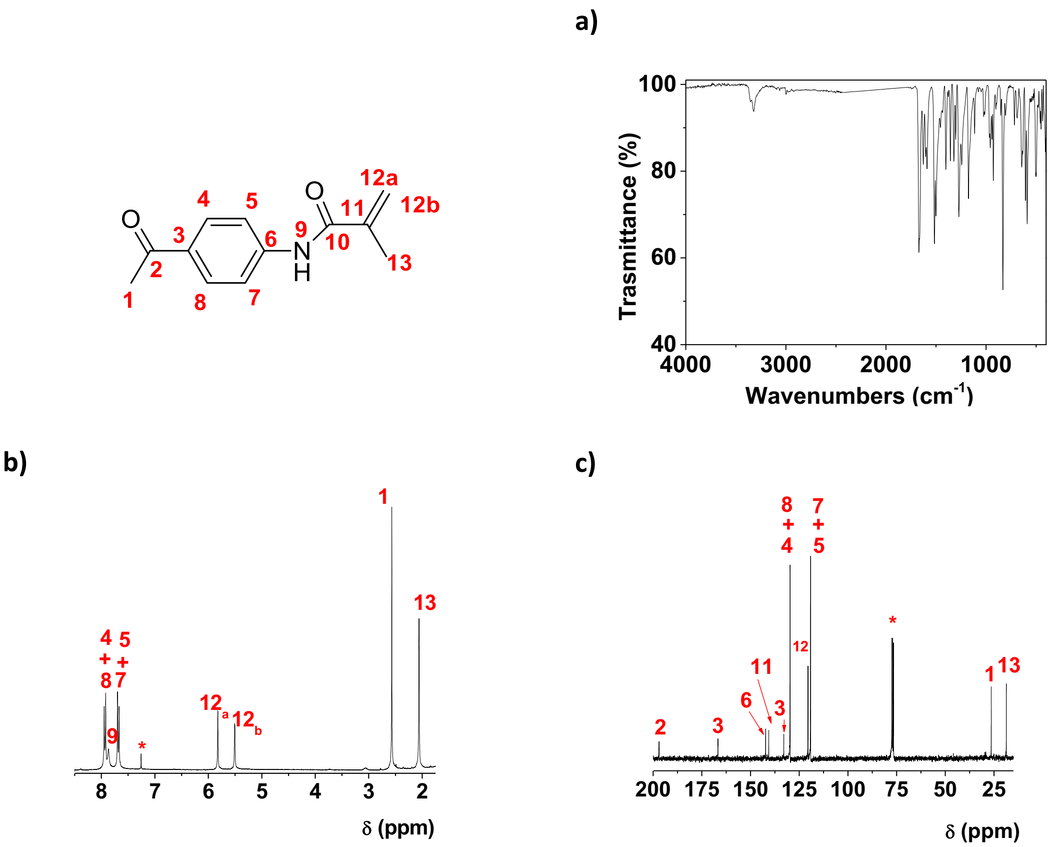

Figure S1. Characterization of *(1)* by (a) FTIR, (b) ^{1}H RMN, and (c) ^{13}C RMN. (* = solvent signal, $CDCl_3$).

- ***Synthesis of N,N'-(((ethane-1,2-diylidenebis(azanylylidene))bis(ethane-1,1-diyl))bis(4,1-phenylene))bis(2-methacrylamide) (2)***

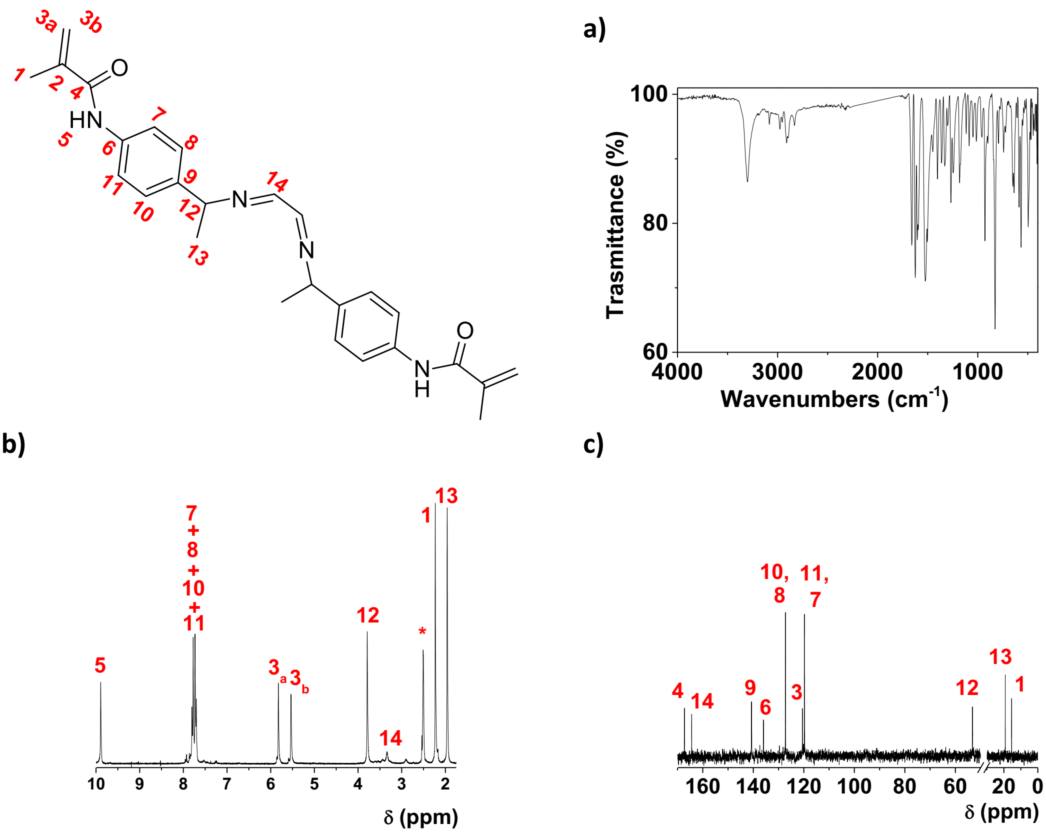

Figure S2. Characterization of *(**2**)* by (a) FTIR, (b) ^{1}H RMN, and (c) ^{13}C RMN. (* = solvent signal, DMSO)

- ***N,N'-(((ethane-1,2-diylbis(azanediyl))bis(ethane-1,1-diyl))bis(4,1-phenylene))bis(2-methacrylamide) (3).***

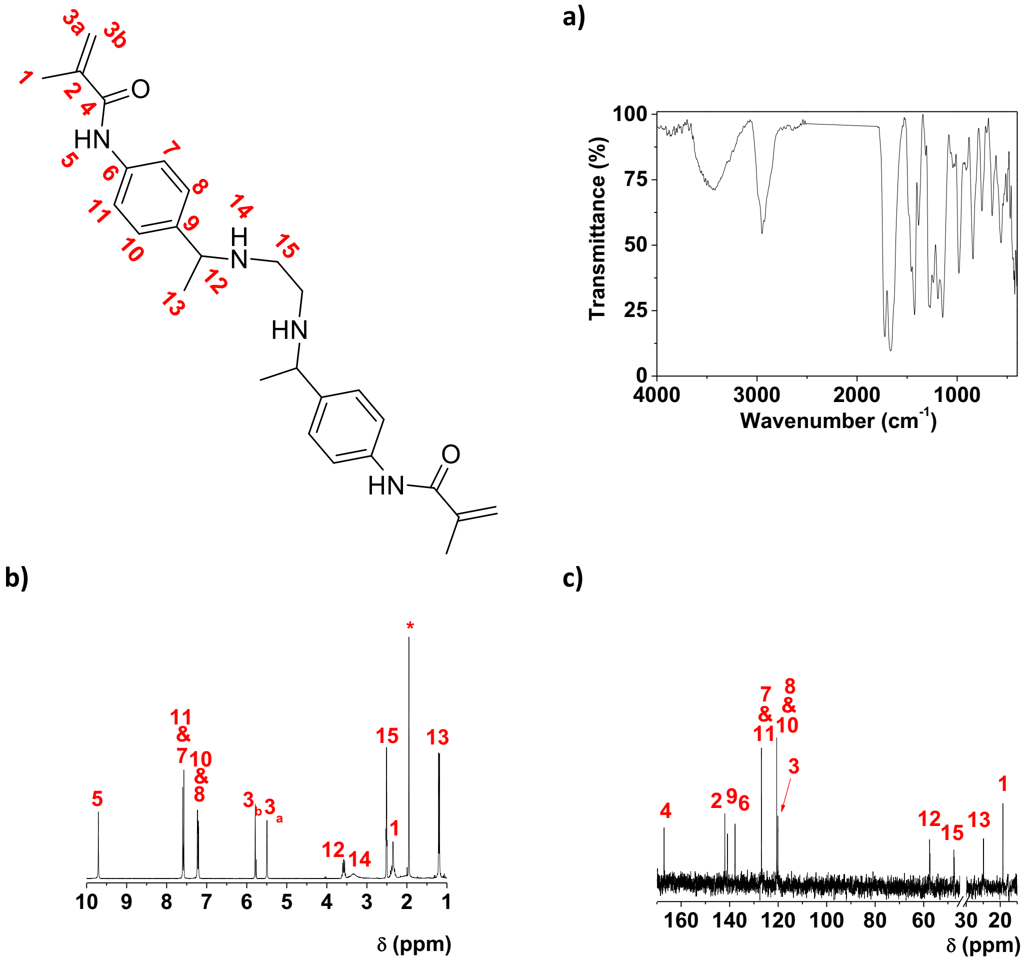

Figure S3. Characterization of *(3)* by (a) FTIR, (b) ^{1}H RMN, and (c) ^{13}C RMN. (* = solvent signal, DMSO)

S2. Thermal characterization of Materials

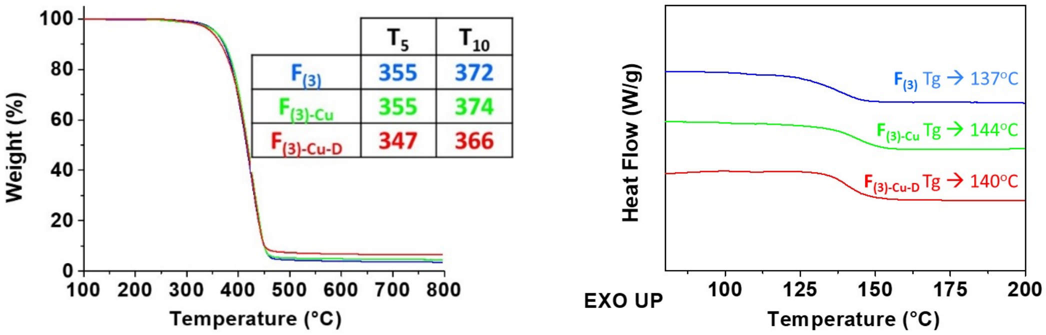

Figure S4. a) Thermogravimetric curves at 10°C/min for $\mathbf{F_{(3)}}$, $\mathbf{F_{(3)\text{-}Cu}}$ and $\mathbf{F_{(3)\text{-}Cu\text{-}D}}$ under nitrogen atmosphere. The figure shows T5 and T10 values; b) DSC curve at 20°C/min for $\mathbf{F_{(3)}}$, $\mathbf{F_{(3)\text{-}Cu}}$ and $\mathbf{F_{(3)\text{-}Cu\text{-}D}}$ under nitrogen atmosphere. The figure shows the glass transition temperature (Tg).

S3. The behaviour of the sensory film at different pH.

The study of the behaviour of the material at different pH was carried out dipping discs of **F$_{(3)\text{-Cu-D}}$** in 5 ml of water solutions with pHs between 0 and 14 overnight. These solutions were prepared from HCl and NaOH in water in different concentrations until the pH range was completed (1-14).

Table S5. RGB data obtained from the digital photos of the **F$_{(3)\text{-Cu-D}}$** discs after immersion in aqueous solutions with different pHs. The table also shows the calculated principal component PC (R&G) parameter.

pH	R	G	B	*PC (R&G)
**1**	194.00	187.00	191.00	2.60
**2**	168.00	151.00	179.00	2.00
**3**	58.00	7.00	172.00	-0.44
**4**	15.00	1.00	185.00	-0.91
**5**	0.00	0.00	183.00	-1.06
**6**	0.00	0.00	187.00	-1.06
**7**	0.00	0.00	186.00	-1.06
**8**	0.00	0.00	178.00	-1.06
**9**	0.00	0.00	184.00	-1.06
**10**	0.00	0.00	183.00	-1.06
**11**	14.00	1.00	181.00	-0.92
**12**	90.00	77.00	192.00	0.54
**13**	134.00	130.00	190.00	1.47
**14**	168.00	151.00	183.00	2.00

* Principal component analysis

Number	Eigenvalue	Variance %	Accumulated %
1	1.98	99.17	99.17
2	$1.66 \cdot 10^{-2}$	0.83	100.00

This procedure runs a principal component analysis. The purpose of the analysis is to obtain a reduced number of linear combinations of the 2 variables that explain the greatest variability in the data. In this case, a component has been extracted, since it is the only component with an eigenvalue greater than or equal to 1, Which explains 99.17% of the variability in the original data.

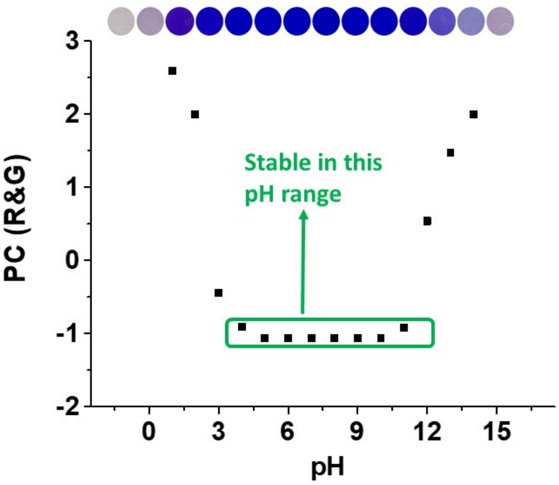

Figure S6. Graphical representation of the calculated principal component of color (obtained from RGB parameters of the digital photographs) versus the pH. The image shows the photographs of the discs after immersion in 5 ml water solutions with pHs between 0 and 14 overnight.

S4. Interference study

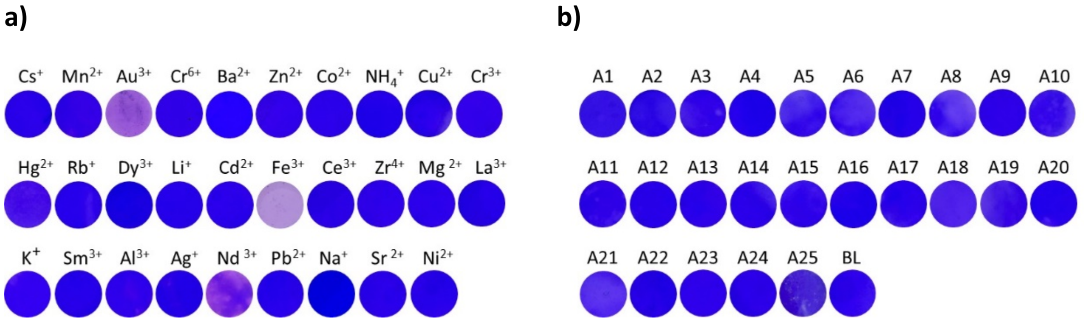

Figure S7. Discs of $F_{(3)\text{-Cu-D}}$ were dipped for 24 h in a mixture of 300 µL of MeOH, 300 µL of pH=7 buffer solution and 30 µL of 0.1 M interferent solution. a) Cations. b) Anions. A1=Cyanide, A2=acetate, A3=hydroxile, A4=fluoride, A5=perchlorate, A6=dodecyl sulfate, A7=nitrite, A8=ethoxide, A9=hydrogen phthalate, A10=pyrophosphate, A11=persulfate, A12=methanesulfonate, A13=pyrophosphate dibasic, A14=trifluoromethanesulfonate, A15=*p*-toluenesulfonate, A16=bromide, A17=thiocyanate, A18=oxalate, A19=carbonate, A20=benzoate, A21=dihydrogenphosphate, A22=sulfate, A23=chloroacetate, A24=trifluoroacetate, A25=periodate.

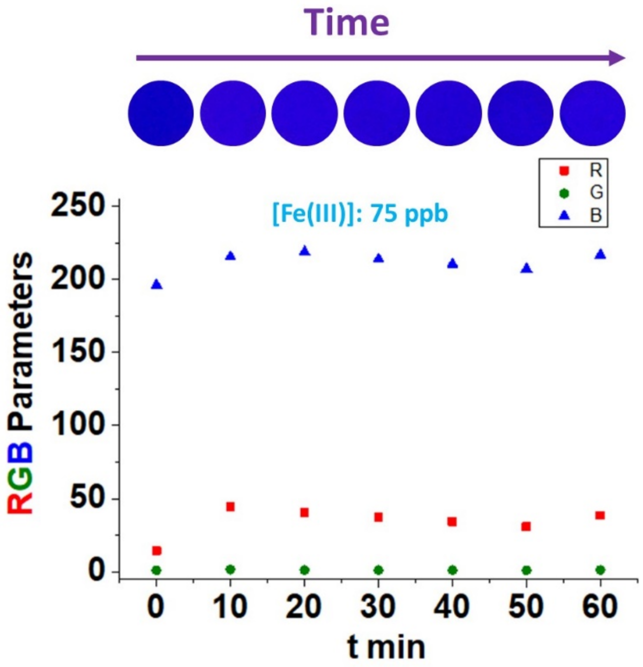

Figure S8. Colour evolution of an $\mathbf{F_{(3)\text{-Cu-D}}}$ disc when dipped for 60 min in a 75 ppb solution of Fe(III) (300 µL of MeOH, 300 µL of pH= 7 buffer solution, and 30 µL of $2.82\text{x}10^{-3}$ M Fe(III) solution). Colour was checked every 10 minutes.

S5. Interaction of the dye with solvents.

Table S9. Obtained wavelengths at the maximum of the UV-Vis curves of **D**, $\mathbf{F_{(3)-D}}$ and $\mathbf{F_{(3)-Cu-D}}$ in different solvents. The table also shows tabulated **π***, α, and β parameters for the Taft-Kamlet solvatochromic model, describing the polarity of the solvent, the acidity or ability to donate a proton to a hydrogen bond (HBD) and the basicity or ability to accept a proton from a hydrogen bond (HBA) respectively.

	D	$F_{(3)-D}$	$F_{(3)-Cu-D}$			
**Solvents**	**λ, nm**	**λ, nm**	**λ, nm**	**π***	**α**	**β**
**1-Butanol**	595.84	598.69	608.58	0.47	0.84	0.84
**EtOH**	584.43	597.77	605.81	0.54	0.86	0.75
**Dioxane**	601.07	597.42	610.44	0.55	0.00	0.37
**AcEt**	591.9	597.00	606.30	0.55	0.00	0.45
**Chloroform**	596.41	595.57	605.03	0.58	0.20	0.10
**THF**	595.65	595.52	606.07	0.58	0.00	0.55
**MeOH**	581.08	594.77		0.60	0.98	0.66
**Acetone**	589.57	592.93	600.39	0.71	0.08	0.43
**Acetonitrile**	585.93			0.75	0.19	0.4
**Nitromethane**	552.33			0.85	0.22	0.06
**Ethylene glycol**		589.26	599.63	0.92	0.90	0.52
**Water**	535.10		597.92	1.09	1.17	0.47

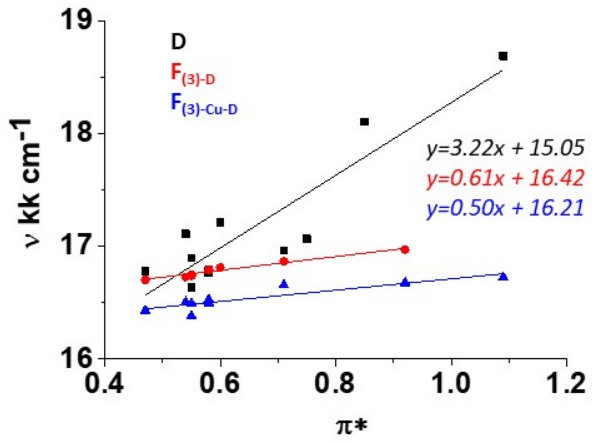

Figure S10. Graphical representation of ν (expressed in 10^3 cm^{-1}, and obtained by dividing by λ) versus the polarity of the solvent (**π***). Black, red and blue lines represent D, $\mathbf{F_{(3)-D}}$, and $\mathbf{F_{(3)-Cu-D}}$ systems respectively.

S6. Diffusion of species in solution into the swelled film.

In this paper, we study the interaction of amino acids with the $F_{(3)\text{-Cu-D}}$ sensor. This substrate is a 100-micron thick membrane, with a receiving unit containing copper attached to a dye, **Figure S11**. The number of receiving units will depend on the weight of the latter used. When the sensor is in contact with a solution of amino acid, adsorption of the latter occurs first. Subsequently, the substitution of the dye by the amino acid takes place, the first one being out of the film and colouring the solution.

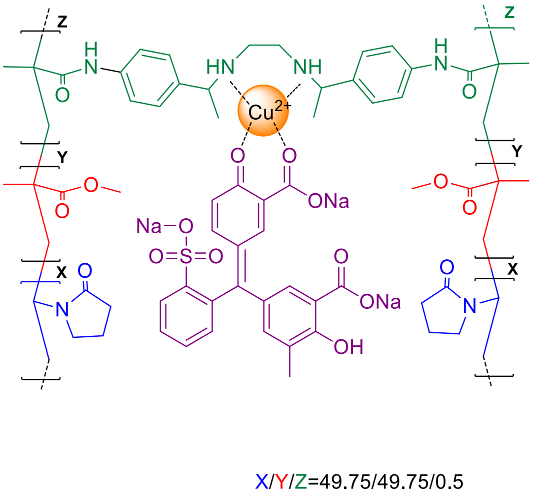

Figure S11. Proposed structure for $\mathbf{F}_{(3)\text{-Cu-D}}$.

To clarify the mechanism that takes place during this process, a kinetic study with several amino acids is carried out. Finally, from an amino acid mixture, the adsorption rate constant is determined to construct a calibration curve and to determine their concentration in a sample of collagen, elastin and epidermis.

There are so many examples in the related literature studying different adsorption processes of substances, generally pollutants, in different substrates with variable particle size. In the present work, the objective is to synthesize an amino acid sensor that will act as an adsorbent. This, instead of being presented in particles of a certain size, is a 100 μm thick membrane.

In the adsorption processes in solution several stages of transport take place in series:

1. External transport of the solute that moves from within the solution to the film surrounding the adsorbent. This is a quick process.
2. Diffusion of the solute through the film towards the surface of the adsorbent or external mass transfer.
3. Intraparticle diffusion.
4. Adsorption itself on the active centres.

There are two approaches in mathematical modelling for the kinetic study of adsorption:

a) Surface reaction model (SRM), where the mass transfer is assumed to be rapid and the adsorption reaction (step 4) is the stage that limits speed.

b) Model of mass transfer reaction (MTM), mass transfer is the slow stage while the adsorption reaction is rapid. In this model, one can find one (single resistance model) or both (dual resistance model) diffusion processes (steps 2 and 3) that control speed.

The obvious drawback of any of the types of modelling (SRM or MTM) is that the apparent numerical value of a speed parameter, obtained by adjusting the model to the data, may not reflect the actual value of the model, but maybe a grouped parameter that incorporates the effect of other processes that were not included in the model derivation. This problem is especially important if the parameter in question (e.g., the diffusion coefficient) needs to have a general validity so that it can be used in different circumstances.

Before the approach of the two models, it is necessary to consider the mass balance, whose differential expression is given by equation (1).

$$V\frac{dC_t}{dt} + M\frac{dq_t}{dt} = 0 \quad (1)$$

Integrating equation (1) and rearranging it gives equation (2).

$$q_t = (C_o - C_t)\frac{V}{M} \quad (2)$$

Where q_t is the milligrams of adsorbate per gram of adsorbent at a time t, C_o and C_t are the milligrams per litre of initial adsorbate and at time t respectively, V is the volume of the solution in litres and M is the grams of the adsorbent.

To calculate q_t, the concentration of adsorbed amino acid must be measured. For this, it is considered that for each mole of adsorbed amino acid one mole of dye is released. The concentration of dye released is determined by measuring the absorbance at each time. Known the molar extinction coefficient at the working wavelength, 431 nm, and the volume of solution, the moles of released dyes that are the same as the adsorbed amino acid are determined. From here, it is possible to calculate q_t at each time.

a) Fixed-bed kinetics: SRM

Several empirical equations allow the study of adsorption kinetics. Two of the most used are the Lagergren [1] equation, pseudo-first-order, and Ho's [2], pseudo-second-order. The theoretical approach of these two models is proposed by Azizian [3], concluding that the obtained speed constants depend on the adsorption rate constants, desorption and the initial concentration of the adsorbate. In the approach developed by Azizian, the cases where the initial adsorbate concentration is much higher than the adsorbed concentration, the equation obtained corresponds to the pseudo-first-order model, equation (3). In this work, the concentration of amino acid in solution practically remains constant, the pseudo-first-order model being the one that best fits the experimental data.

$$q_t = q_e(1 - e^{-k_1 t}) \quad (3)$$

Where q_e are the milligrams adsorbed when equilibrium is reached, and the speed constant of the process is reached. From a non-linear adjustment by least squares, these parameters are determined. **Figure S12** shows the adjustment obtained for one of the amino acids analysed.

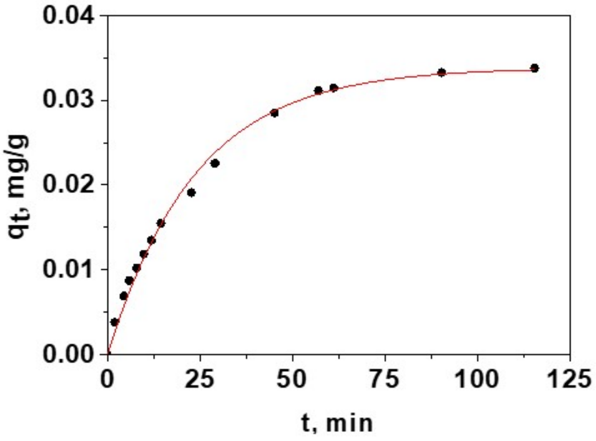

Figure S12. Surface reaction model for glycine.

The results obtained for the different amino acids studied are shown in **Table S13**.

Table S13. Kinetic parameters obtained by non-linear adjustment by least squares to equation (3). [Amino acid]$_o$ = 4.76x10^{-4} M.

Amino acid	q_e, mg g^{-1}	k_1, min^{-1}	R
**Arginine**	0.128 ± 0.008	0.005 ± 0.001	0.9978
**Aspartic Acid**	0.105 ± 0.001	0.010 ± 0.001	0.9973
**Phenylalanine**	0.160 ± 0.010	0.014 ± 0.001	0.9987
**Glutamic Acid**	0.088 ± 0.001	0.018 ± 0.001	0.9989
**Hydroxyproline**	0.086 ± 0.002	0.023 ± 0.002	0.9970
**Proline**	0.074 ± 0.001	0.026 ± 0.001	0.9962
**Alanine**	0.057 ± 0.001	0.034 ± 0.001	0.9981
**Valine**	0.062 ± 0.001	0.039 ± 0.001	0.9976
**Glycine**	0.034 ± 0.001	0.042 ± 0.002	0.9971

b) Batch kinetics: MTM.

In this mathematical model, there can be up to two stages that control the kinetic process. Therefore, it is necessary to first consider the two types of transfer: diffusion through external film and intraparticle diffusion.

The diffusion through the external film is modelled according to a linear driving force that assumes that the mass transfer rate depends linearly on the difference in concentration between the concentration in solution and the concentration on the external surface of the adsorbent:

$$\frac{\partial q_t}{\partial t} = k_f S_A (C_t - C_i) \quad (4)$$

Where k_f is the external mass or film transfer coefficient, S_A is the specific surface area of the adsorbent, C_t and C_i are the concentrations of the adsorbate in the solution at a time t and on the surface in equilibrium with the phase concentration solid, q.

For intraparticle diffusion, the model assumes a uniform membrane of thickness l and that the solute diffuses through it with a constant diffusivity Ds. Fick's law of diffusion for this system is given by equation (5).

$$\frac{\partial q_t}{\partial t} = D_s \left(\frac{\partial^2 q_t}{\partial x^2} \right) \quad (5)$$

b1. Single resistance: intraparticle-diffusion models

In this model, it is assumed that diffusion occurs in a homogeneous solution of limited volume. The membrane is considered to have a thickness 2l, where the space occupied by it is $-l \leq x \leq l$, while the solution is limited to space $-l-a \leq x \leq -l$, $l \leq x \leq l + a$. The concentration of the solute in the solution is always uniform and is initially C_o, while in the membrane the initial concentration is zero. Crank provides the analytical solution [4], assuming that intraparticle diffusion is the only stage of speed control.

$$\frac{q_t}{q_e} = 1 - \sum_{n=1}^{\infty} \frac{2\alpha(1+\alpha)}{1+\alpha+\alpha^2\gamma_n^2} \exp\left(-\frac{D_s \gamma_n^2 t}{l^2}\right) \quad (6)$$

Where γ_n is the non-zero positive roots of:

$$tan\, \gamma_n = -\alpha \ \gamma_n \quad (7)$$

The parameter α is the relationship between the volume of the solution and the membrane and can be calculated employing equation (8)

$$\frac{Mq_e}{2aC_o} = \frac{1}{1+\alpha} \quad (8)$$

Calculated α, the values of γ_n are determined and by a non-linear adjustment by least-squares D_s is optimized. **Figure S14** shows the adjustment obtained for one of the amino acids. This model has been used by other researchers for spherical particles [5].

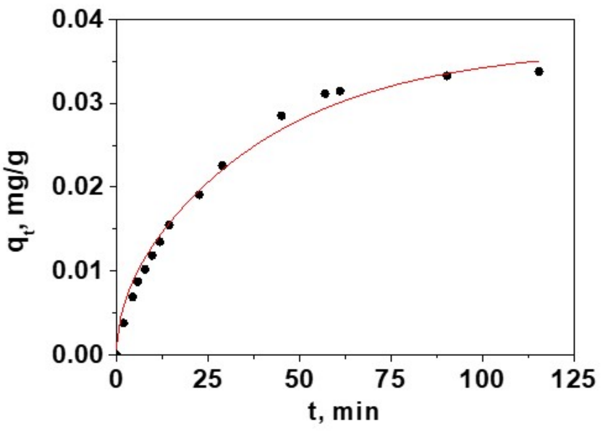

Figure S14: Crank model of intraparticle diffusion for glycine.

Following the results obtained, **Figure S14**, suggests that, although intraparticle diffusion is important, it is the two stages that intervene in the adsorption process, without ruling out that it is intraparticle diffusion that controls speed.

b2. Single resistance: film-diffusion models.

Considering a finite volume, equation (2) and a linear isotherm (q_e = KC_e) (where C_i is in equilibrium with q as C_e with q_e) C_t and C_i can be substituted in equation (4) in terms of C_o and q_t to obtain equation (9), which can be integrated giving rise to equation (10).

$$\frac{dq_t}{dt} = k_f S_A \left(C_o - q\left(\frac{M}{V} + \frac{1}{K}\right)\right) \qquad (9)$$

$$\frac{q_t}{q_e} = 1 - \exp\left(-k_f S_A \left(\frac{MK + V}{VK}\right) t\right) \qquad (10)$$

The exponential term in equation (10) is constant for any set of experimental data, and this is equivalent to equation (3). Comparing both equations, it can be argued that the kinetic rate constant k_1 is a function of S_A, K, M, V and k_f. On the other hand, the empirical model of Boyd [6] and Reichenberg [7] used to study intraparticle diffusion also establishes a linear relationship between $-\ln\left(1 - {q_t}/{q_e}\right)$ and time, equation (11).

$$Bt = -ln\left(1 - \frac{q_t}{q_e}\right) - 0.4977 \qquad (11)$$

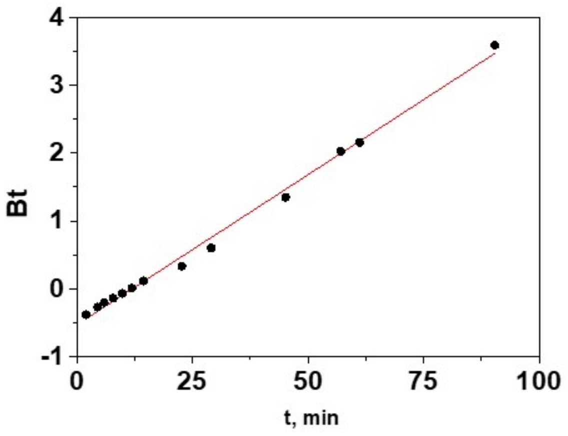

Figure S15: Boyd's model for Glycine

Figure S15 shows the result of applying the Boyd model. This mathematical analogy means that a linear relationship between$-\ln\left(1 - {q_t}/{q_e}\right)$ and time can be derived from three opposite assumptions: surface reaction model (SRM) equation (3) of pseudo-first-order, intraparticle diffusion equation (11) and diffusion through the external film equation (10). Although the experimental data conform to equation (10), (the same result is obtained as with equation (3), **Figure S12**), it is not conclusive given all the above, but the assumption that the diffusion of the film is the stage of speed control.

b3. Dual resistance model (intraparticle and film diffusion)

Crank [4] derived the model analytically with a more realistic solution that includes both broadcasts, equation (12).

$$\frac{q_t}{q_e} = 1 - \sum_{n=1}^{\infty} \frac{2B_i^2 exp(-\beta_n^2 D_s t/l^2)}{\beta_n^2(\beta_n^2 + B_i^2 + B_i)} \qquad (12)$$

Where βn are the positive roots of:

$$\beta_n tan\beta_n = B_i \qquad (13)$$

The B_i parameter depends in turn on k_f, D_S and l, equation (14),

$$B_i = \frac{k_f l}{D_s} \qquad (14)$$

With equations (12) to (14) and using the Origin program, the parameters Ds and k_f were optimized by a non-linear adjustment by least squares. **Figure S16** shows the results obtained for some of the amino acids. This model, for spherical particles, has been used by other researchers [8,9].

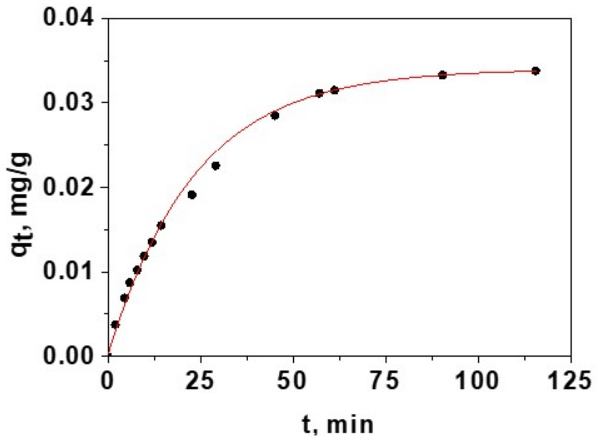

Figure S16: Crank dual resistance model for Glycine.

From the results obtained, it is confirmed that the two diffusion processes are involved in the adsorption reaction. **Table 2** (manuscript) shows the parameters obtained in the adjustment with equation (12).

Once the external and internal diffusion coefficients for a given adsorption system have been determined, the speed limitation step can be determined in terms of the number of Biot, B_i, which relates the external mass transfer resistance to the resistance of internal mass transfer, equation (14). When the B_i >> 1, the adsorption process is mainly controlled by intraparticle diffusion, and if Bi<< 1, it is the external diffusion that primarily controls the speed [10,11]. Once the values of B_i have been checked, table 2, the stage that mainly controls this adsorption process is the diffusion on the boundary layer or external mass transport.

S7. Complex formation between the polymer, Cu(II) and dye.

The complex that forms within the film is composed of 1 metal centre (Copper) and two different types of ligands: the compound (3) and the dye.

We have tried to see the complex formed between (3) and Cu(II), but we have not been able to absorb it in the same area. However, we assume that the complex within the membrane is 1: 1 since it is prepared by immersing the material in a brutal excess of copper.

In the second step, the film is immersed in a dye solution. In this case, the 1:1 complex is also going to be formed for the same reason as before.

Therefore, we prepare an assessment in which we start from dye, and we are adding increasing amounts of a mixture of (3) and Cu(II) in 1: 1 molar ratio, using pure DMA as the solvent. Results are shown in **Figure S17**, where an isosbestic point around 350 nm can be seen perfectly. We can separate the graphs into three different processes attending to the shape of the curves, i.e., the formation of the complexes 1:1, 2:1 and 3:1.

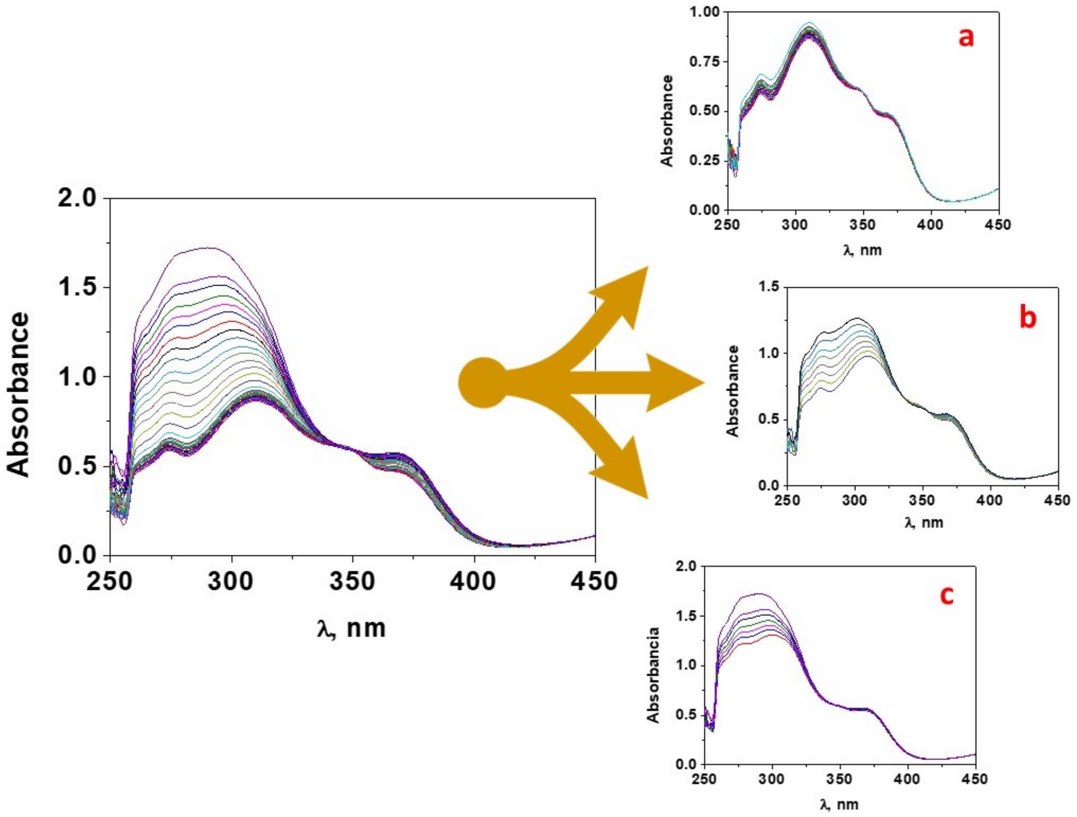

Figure S17. Titration of {(3)-Cu(II)} (1:1) with D in DMA as the solvent, for concentrations of {(3)-Cu(II)} (1:1) ranging from 9.5x10^{-7} M to 3.42x10^{-4} M. Initial concentration of D: 1.9x10^{-5} M. **a**, **b** and **c** graphs show the formation processes of three different complexes between {(3)-Cu(II)} and D, with stoichiometries 1:1, 2:1 and 3:1 respectively.

Additionally, Job´s plot diagram was calculated and represented in **Figure S18.**

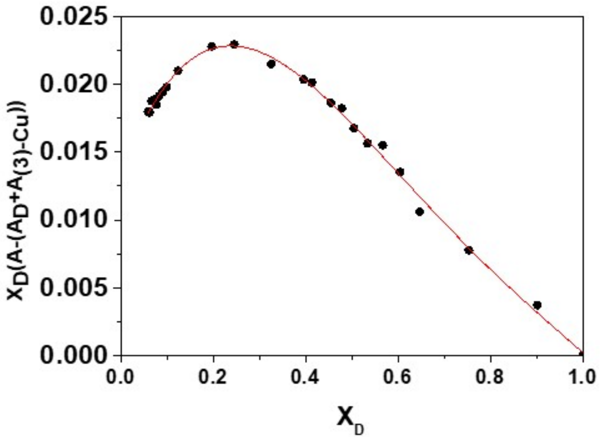

Figure S18. Graphical representation of the parameter X_D·(A-(A_D+$A_{(3)-Cu}$)) versus X_D. The maximum at 0.25 X_D means that the formed complex has a 3:1 stoichiometry.

S8. Preparation of the solutions mimicking collagen, elastin, and epidermis.

Table S19. Prepared solution mimicking collagen in different concentrations (**A** to **G**).

	Solutions						
	**A**	**B**	**C**	**D**	**E**	**F**	**G**
**Glycine**	$1.77x10^{-3}$	$8.85x10^{-4}$	$1.77x10^{-4}$	$8.84x10^{-5}$	$1.77x10^{-5}$	$8.84x10^{-6}$	$1.77x10^{-6}$
**Proline**	$6.21x10^{-4}$	$3.11x10^{-4}$	$6.21x10^{-5}$	$3.11x10^{-5}$	$6.21x10^{-6}$	$3.11x10^{-6}$	$6.21x10^{-7}$
**Hidroxiproline**	$5.18x10^{-4}$	$2.59x10^{-4}$	$5.19x10^{-5}$	$2.59x10^{-5}$	$5.18x10^{-6}$	$2.59x10^{-6}$	$5.18x10^{-7}$
**Glutamic Acid**	$3.86x10^{-4}$	$1.93x10^{-4}$	$3.86x10^{-5}$	$1.93x10^{-5}$	$3.86x10^{-6}$	$1.93x10^{-6}$	$3.86x10^{-7}$
**Alanine**	$6.04x10^{-4}$	$3.02x10^{-4}$	$6.04x10^{-5}$	$3.02x10^{-5}$	$6.04x10^{-6}$	$3.02x10^{-6}$	$6.04x10^{-7}$
**Arginine**	$6.10x10^{-4}$	$3.05x10^{-4}$	$6.10x10^{-5}$	$3.05x10^{-5}$	$6.10x10^{-6}$	$3.05x10^{-6}$	$6.10x10^{-7}$
**Aspartic Acid**	$2.53x10^{-4}$	$1.27x10^{-4}$	$2.53x10^{-5}$	$1.27x10^{-5}$	$2.53x10^{-6}$	$1.27x10^{-6}$	$2.53x10^{-7}$
Σ molarities	$4.76x10^{-3}$	$2.38x10^{-3}$	$4.76x10^{-4}$	$2.38x10^{-4}$	$4.76x10^{-5}$	$2.38x10^{-5}$	$4.76x10^{-6}$

Table S20. Prepared solution mimicking elastin in different concentrations (**A** to **E**)

	Solutions				
	**A**	**B**	**C**	**D**	**E**
**Glycine**	$1.11x10^{-3}$	$2.22x10^{-4}$	$1.11x10^{-4}$	$5.56x10^{-5}$	$2.22x10^{-5}$
**Alanine**	$7.01x10^{-4}$	$1.40x10^{-4}$	$7.01x10^{-5}$	$3.51x10^{-5}$	$1.40x10^{-5}$
**Valine**	$3.14x10^{-4}$	$6.29x10^{-5}$	$3.14x10^{-5}$	$1.57x10^{-5}$	$6.29x10^{-6}$
**Proline**	$2.53x10^{-4}$	$5.06x10^{-5}$	$2.53x10^{-5}$	$1.26x10^{-5}$	$5.06x10^{-6}$
Σ molarities	$2.38x10^{-3}$	$4.76x10^{-4}$	$2.38x10^{-4}$	$1.19x10^{-4}$	$4.76x10^{-5}$

Table S21. Prepared solution mimicking epidermis in different concentrations (**A** to **G**).

	Solutions			
	**A**	**B**	**C**	**D**
**Hydroxyproline**	$3.34x10^{-4}$	$2.50x10^{-4}$	$1.67x10^{-4}$	$3.34x10^{-5}$
**Aspartic acid**	$1.71x10^{-4}$	$1.28x10^{-4}$	$8.53x10^{-5}$	$1.71x10^{-5}$
**Threonine**	$6.88x10^{-5}$	$5.16x10^{-5}$	$3.44x10^{-5}$	$6.88x10^{-6}$
**Serine**	$1.69x10^{-4}$	$1.27x10^{-4}$	$8.45x10^{-5}$	$1.69x10^{-5}$
**Glutamic acid**	$2.65x10^{-4}$	$1.99x10^{-4}$	$1.33x10^{-4}$	$2.65x10^{-5}$
**Proline**	$5.10x10^{-4}$	$3.83x10^{-4}$	$2.55x10^{-4}$	$5.10x10^{-5}$
**Glycine**	$2.13x10^{-3}$	$1.60x10^{-3}$	$1.06x10^{-3}$	$2.13x10^{-4}$
**Alanine**	$5.93x10^{-4}$	$4.45x10^{-4}$	$2.97x10^{-4}$	$5.93x10^{-5}$
**Cysteine**	$2.19x10^{-6}$	$1.64x10^{-6}$	$1.09x10^{-6}$	$2.19x10^{-7}$
**Valine**	$1.02x10^{-4}$	$7.63x10^{-5}$	$5.09x10^{-5}$	$1.02x10^{-5}$
**Methionine**	$2.05x10^{-5}$	$1.54x10^{-5}$	$1.03x10^{-5}$	$2.05x10^{-6}$
**Isoleucine**	$4.12x10^{-5}$	$3.09x10^{-5}$	$2.06x10^{-5}$	$4.12x10^{-6}$
**Leucine**	$8.98x10^{-5}$	$6.73x10^{-5}$	$4.49x10^{-5}$	$8.98x10^{-6}$
**Tyrosine**	$1.04x10^{-5}$	$7.76x10^{-6}$	$5.18x10^{-6}$	$1.04x10^{-6}$
**Phenylalanine**	$3.80x10^{-5}$	$2.85x10^{-5}$	$1.90x10^{-5}$	$3.80x10^{-6}$
**Lysine**	$8.59x10^{-5}$	$6.45x10^{-5}$	$4.30x10^{-5}$	$8.59x10^{-6}$
**Histidine**	$1.17x10^{-5}$	$8.79x10^{-6}$	$5.86x10^{-6}$	$1.17x10^{-6}$
**Arginine**	$1.21x10^{-4}$	$9.04x10^{-5}$	$6.03x10^{-5}$	$1.21x10^{-5}$
Σ molarities	$4.76x10^{-3}$	$3.58x10^{-3}$	$2.38x10^{-3}$	$4.76x10^{-4}$

S9. Kinetic constants for collagen, elastin and epidermis and different *Σ molarities (Σ M)*.

Table S22. Kinetics constants for collagen

Solutions	*Σ M*, COL	k_1	R
**A**	4.76x10^{-3}	0.145 ± 0.007	0.9972
**B**	2.38x10^{-3}	0.079 ± 0.003	0.9965
**C**	4.76x10^{-4}	0.014 ± 0.001	0.9947
**D**	2.38x10^{-4}	0.010 ± 0.001	0.9975
**E**	4.76x10^{-5}	0.006 ± 0.001	0.9959
**F**	2.38x10^{-5}	0.006 ± 0.001	0.9906
**G**	4.76x10^{-6}	0.005 ± 0.001	0.9990

Table S23. Kinetics constants for elastin

Solutions	*Σ M*, ELA	k_1	R
**A**	2.38x10^{-3}	0.096 ± 0.002	0.9989
**B**	4.76x10^{-4}	0.024 ± 0.001	0.9981
**C**	2.38x10^{-4}	0.010 ± 0.001	0.9994
**D**	1.19x10^{-4}	0.006 ± 0.001	0.9989
**E**	4.76x10^{-5}	0.008 ± 0.001	0.9961

Table S24. Kinetics constants for the epidermis

Solutions	*Σ M*, EPI	k_1	R
**A**	4.76x10^{-3}	0.170±0.010	0.9954
**B**	3.57x10^{-3}	0.127±0.005	0.9977
**C**	2.38x10^{-3}	0.091±0.007	0.9943
**D**	4.76x10^{-4}	0.021±0.001	0.9972

S10. Calculation of initial rates at 1 and 5 min.

As previously seen, the most accurate is to measure the reaction rate constant. However, depending on the concentration this measure could last for hours, so we think about using that same philosophy (measuring the kinetics) but in short times.

To obtain an easy-to-use calibration curve, the value of the initial rate in the first minutes of the reaction is determined and plotted against the glycine concentration. The initial rate was obtained from the origin of the graphical representation of absorbance/time versus time. This linear trend is only maintained during the first minutes of the adsorption process, thus, we have calculated the value of the initial rate with the absorbance data at 1 and 5 minutes by equation (16).

$$v_o = \frac{A_5}{5} - \left(\frac{\frac{A_5}{5} - \frac{A_1}{1}}{5-1}\right)5 \quad (16)$$

Calculated the initial rate and represented against the concentration of glycine, **Figure S25** is obtained. A linear variation is observed, with ordinate (1.2±0.2) 10^{-3} min^{-1}, slope 23.8±0.3 min^{-1} M^{-1} and a value of R = 0.9992.

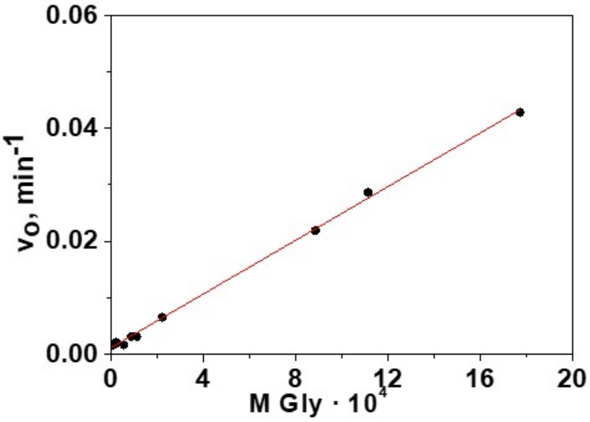

Figure S25: Graphical representation of the initial rates versus glycine concentration

To determine the concentration of an unknown sample, a sensor disc is introduced to a solution of this and measurements are taken at one minute (A1) and five minutes (A5). With the help of equation (7), the initial rate, vo. To determine the concentration, equation (8) is used.

$$C_{Gly} = \frac{v_o - 0.0012}{23.8} \quad (8)$$

Once the concentration of Glycine, C_{Gly}, and taking into account the steps taken to prepare these samples, the starting concentration, C_p, is determined as $C_p = 21\ C_{Gly}$. To calculate the mass, m, in milligrams of this initially present m = 75.07 C_p V, where V is the ml of starting solution.

This last method depends on the membrane used, so before using it would be necessary to perform a pre-calibration for each of these.

Once tested for glycine, we tested it for the epidermis. The results are shown in **Table S26** and **Figure S27**.

Table S26. The absorbance of at 431 nm of solutions mimicking epidermis at different concentrations after dipping a 10 mm $\mathbf{F_{(3)\text{-}Cu\text{-}D}}$ disc. The absorbance was measured every minute for the 5 minutes.

	**Time (min)**						
**Solutions**	**Σ M**	**0**	**1**	**2**	**3**	**4**	**5**
**A**	$4.76 \cdot 10^{-3}$	$-8.00 \cdot 10^{-3}$	$-5.00 \cdot 10^{-3}$	0.15	0.21	0.24	0.25
**B**	$3.57 \cdot 10^{-3}$	$-3.00 \cdot 10^{-3}$	$4.90 \cdot 10^{-2}$	0.12	0.14	0.17	0.21
**C**	$2.38 \cdot 10^{-3}$	$2.00 \cdot 10^{-3}$	$4.50 \cdot 10^{-2}$	0.08	0.11	0.12	0.15
**D**	$1.19 \cdot 10^{-3}$	$-3.10 \cdot 10^{-2}$	$-6.00 \cdot 10^{-3}$	0.01	0.02	0.05	0.10
**E**	$4.76 \cdot 10^{-4}$	$6.00 \cdot 10^{-3}$	$1.20 \cdot 10^{-2}$	0.01	0.02	0.03	0.04

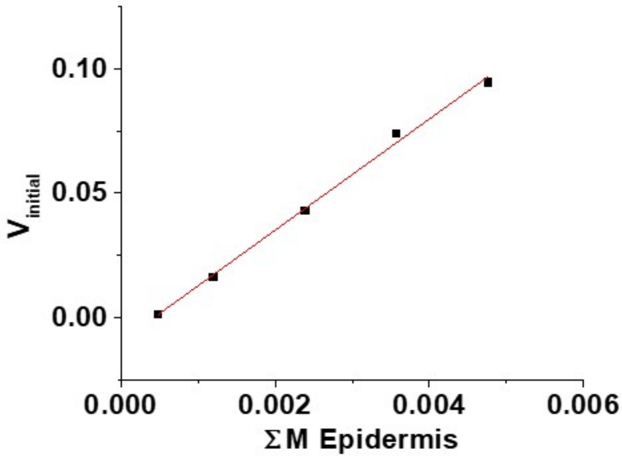

Figure S27. Graphical representation of the initial rates versus ***Σ molarity*** of epidermis.

S11. RGB method

a)

	**M Gly**	**R**	**G**	**B**	***PC (R&G)**
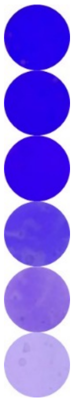	$1.00x10^{-6}$	36	1	239	-1.15
	$1.00x10^{-5}$	40	1	234	-1.10
	$1.00x10^{-4}$	51	1	237	-0.98
	$5.00x10^{-4}$	94	64	231	0.09
	$1.00x10^{-3}$	139	102	228	0.94
	$5.00x10^{-3}$	190	170	242	2.14

* Principal component analysis

**Number**	**Eigenvalue**	**Variance %**	**Accumulated %**
1	1.99	99.75	99.75
2	$5.01·10^{-3}$	$2.51·10^{-1}$	100.00

This procedure runs a principal component analysis. The purpose of the analysis is to obtain a reduced number of linear combinations of the 2 variables that explain the greatest variability in the data. In this case, a component has been extracted, since it is the only component with an eigenvalue greater than or equal to 1.0. Which explains 99.7493% of the variability in the original data.

b)

Equation → y = START + (END - START) * x^n / (k^n + x^n)

R^2 → 0.99969

	Value	Standard Error
START	-1.13	$1.60x10^{-2}$
END	2.34	$4.30x10^{-2}$
k	$7.66x10^{-4}$	$1.91x10^{-5}$
n	1.48	$6.20x10^{-2}$

Figure S28. a) RGB and PC(R&G) data for the titration of glycine with the RGB method. The Figure shows the photographs of $\mathbf{F_{(3)\text{-}Cu\text{-}D}}$ discs after immersion for 60 min in 0.63 ml of MeOH: pH 7 buffer solution (1:1) for concentrations of glycine ranging from $1x10^{-6}$ M to $5x10^{-3}$ M. **b)** Fitted curve.

a)

	Σ M, EPI	R	G	B	*PC (R&G)
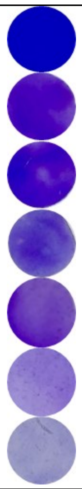	1.00x10^{-2}	0	0	188	-2.26
	1.50x10^{-2}	72	5	187	-1.23
	2.00x10^{-2}	67	12	191	-1.22
	2.50x10^{-2}	86	59	188	-0.45
	3.30x10^{-2}	101	69	185	-0.14
	4.20x10^{-2}	145	126	207	1.08
	5.00x10^{-2}	151	150	196	1.42

* Principal component analysis

Number	Eigenvalue	Variance %	Accumulated %
1	1.91	95.35	95.35
2	9.30·10^{-2}	4.65	100.00

This procedure runs a principal component analysis. The purpose of the analysis is to obtain a reduced number of linear combinations of the 2 variables that explain the greatest variability in the data. In this case, a component has been extracted, since it is the only component with an eigenvalue greater than or equal to 1.0. Which explains 95.35% of the variability in the original data.

b)

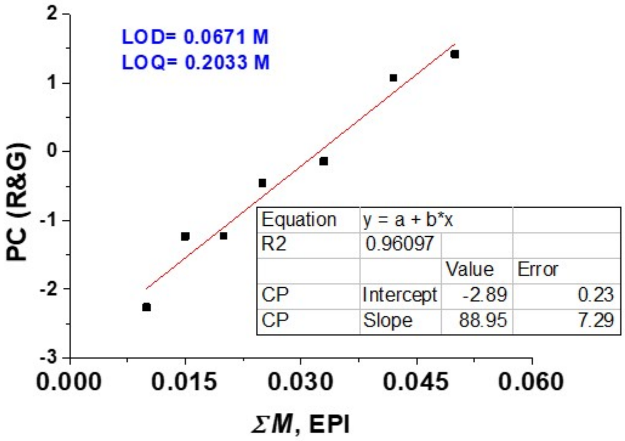

Figure S29. a) RGB and PC(R&G) data for the titration of the epidermis with the RGB method. The Figure shows the photographs of **$F_{(3)\text{-}Cu\text{-}D}$** discs after immersion for 5 min in 0.63 ml of MeOH : pH 7 buffer solution (1:1) for *Σ M* of epidermis ranging 0.01 M to 0.05 M. **b)** Fitted curve, and obtained limits of detection (LOD) and quantification (LOQ).

a)

	ΣM, EPI	R	G	B	*PC(R&G)
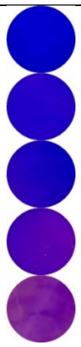	$1.00x10^{-2}$	0	0	203	-1.46
	$2.50x10^{-2}$	38	1	200	-0.81
	$5.00x10^{-2}$	23	0	187	-0.52
	$7.50x10^{-2}$	86	1	176	0.79
	$10.00x10^{-2}$	123	35	158	2.01

* Principal component analysis

Number	Eigenvalue	Variance %	Accumulated %
1	1.79	89.53	89.53
2	0.21	10.47	100.00

This procedure runs a principal component analysis. The purpose of the analysis is to obtain a reduced number of linear combinations of the 2 variables that explain the greatest variability in the data. In this case, a component has been extracted, since it is the only component with an eigenvalue greater than or equal to 1.0. Which explains 89.5322% of the variability in the original data.

b)

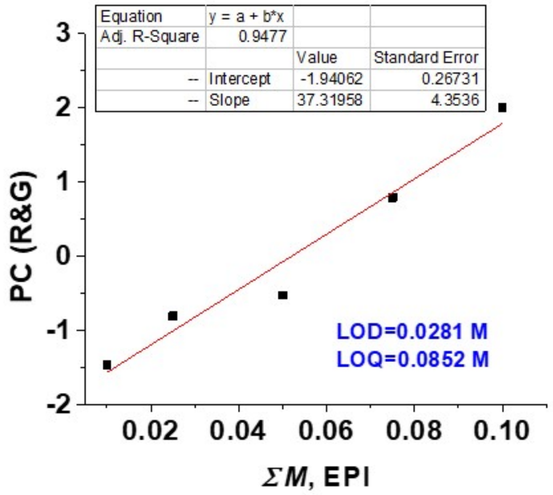

Figure S30. a) RGB and PC(R&G) data for the titration of the epidermis with the RGB method. The Figure shows the photographs of $\mathbf{F}_{(3)\text{-Cu-D}}$ discs after immersion for 1 min in 0.63 ml of MeOH : pH 7 buffer solution (1:1) for ΣM of epidermis ranging 0.01 M to 0.1 M. **b)** Fitted curve, and obtained limits of detection (LOD) and quantification (LOQ).

S12. Proof of concept 1. Hydrolysis of a sample of food matrix (beef, loin cut).

- **Reference method** [12]**.**

OPA solution was prepared by mixing 2.5 mL of 20 % aqueous solution of SDS (sodium dodecyl sulfate), 20 mL of aqueous sodium tetraborate 0.1 M, 160 mg of *o*-phthaldialdehyde (OPA) in 4 mL of ethanol and 0.4 mL of 2-Mercaptoethanol. The solution was made up to 200 mL with deionized water.

To prepare the sample, 12.5 g of the food matrix showed in **Figure S31** (5% substrate to the total volume) and 62.5 mg of the enzyme "papain" (0.5% to the substrate) were weighed and made up to 250 ml with pH 7 buffer solution. The mixture was incubated at 50ºC, and aliquots of 5 mL were taken at t= 21, 40, 62, 120, 180, 240, 300, 360, 420, 480, 540 and 5772 minutes. These aliquots were boiled at 100ºC for 10 min to stop hydrolysis. Finally, they were hot filtered.

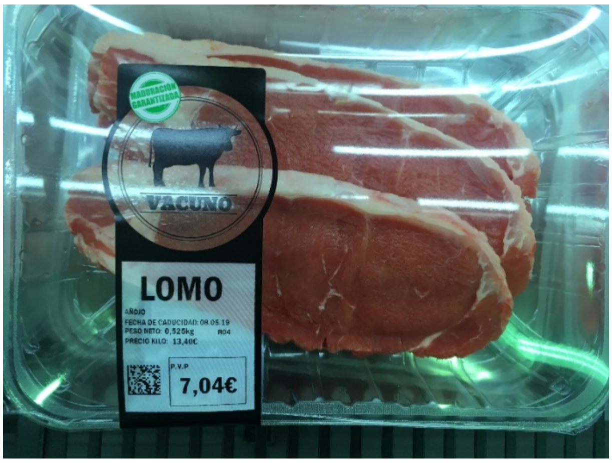

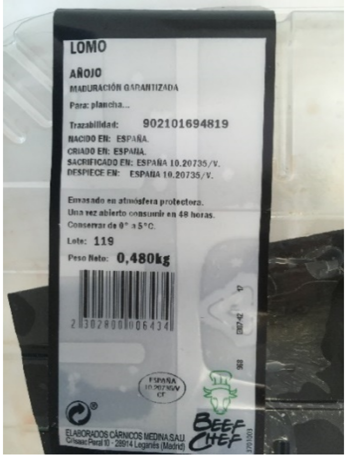

Figure S31. Food matrix employed for the proof of concept.

Finally, 50 μl of the filtered solutions were mixed at room temperature with 3 ml of OPA solution within the cuvette. The mixture stood for exactly 2 min before being read at 330 nm in the spectrophotometer. Each sample is measured by triplicate.

The degree of hydrolysis (DH%) under the following formula.

$$DH\% = \frac{A_{sample} - A_{blank}}{A_{100\%} - A_{blank}} \times 100 \qquad (9)$$

The samples were simultaneously measured with our proposed material, by UV-Vis technique and RGB method.

- **Initial rates (1 min & 5 min) method:**

DH%	Time (min)	v_o
8.00	40	
10.67	62	$9.60 \cdot 10^{-3}$
18.06	120	$1.14 \cdot 10^{-2}$
24.28	180	$1.24 \cdot 10^{-2}$
31.86	240	$1.48 \cdot 10^{-2}$
36.92	300	$1.52 \cdot 10^{-2}$
42.30	360	$1.41 \cdot 10^{-2}$
45.61	420	$1.57 \cdot 10^{-2}$
52.40	540	$1.67 \cdot 10^{-2}$
100	5772	$2.50 \cdot 10^{-2}$

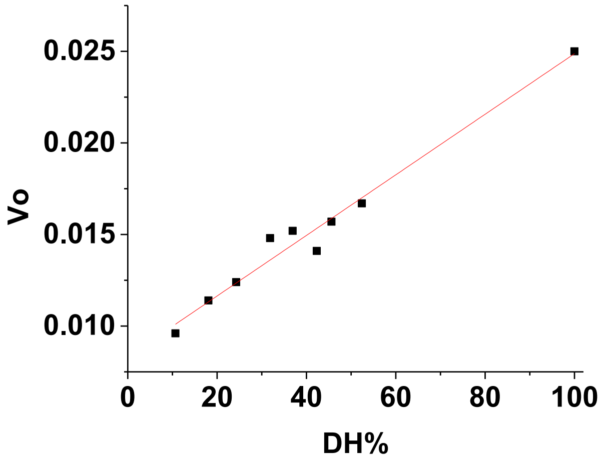

Figure S32. Graphical representation of initial rates versus the degree of hydrolysis (DH%)

S13. Proof of concept 2. Biological samples from a chronic wound.

Different types of samples (**Figure S33**) from the same chronic wound were analysed. Each sample was boiled in a pH 7 buffer solution for 10 min (20 ml of pH 7 buffer solution per gram of sample). Finally, the samples were hot filtered.

Sample	Type of Sample	Weight (mg)	ABS 330 nm (Ref. method)	k_2*
**A**	Swab	47	$5.50x10^{-2}$	-
**B**	Wound bed	148	$4.20x10^{-2}$	0.08272
**C**	Edge	122	$6.70x10^{-2}$	0.15244
**D**	Capsular tissue	160	$3.40x10^{-2}$	0.06152
**E**	Bone	179	$3.20x10^{-2}$	0.05302

*We have worked with a pseudo-second-order kinetic equation (k_2) for modelling this system. The difference with the proposed pseudo-first-order kinetic equation (k_1) in the manuscript lies in the concentration of glycine, which in this case, seems to be quite lower.[¡Error! Marcador no definido.]

a) **b)**

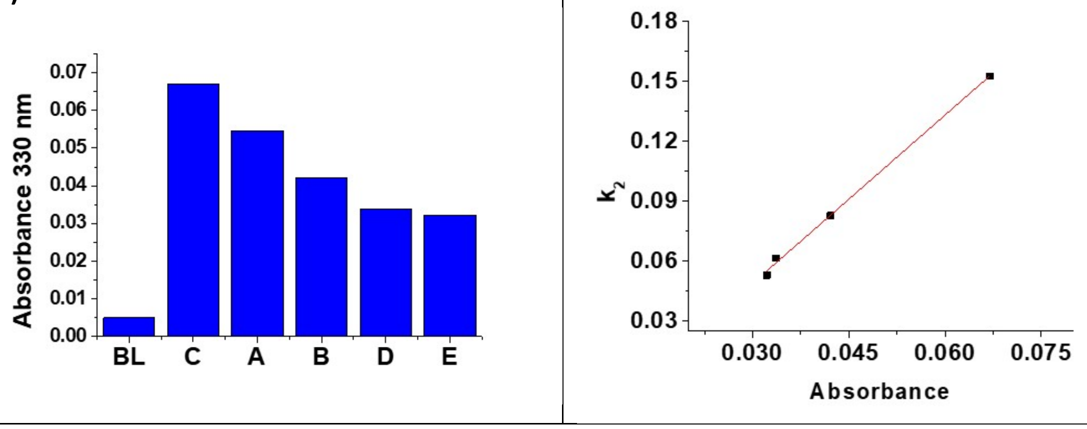

Figure S33. Information about biological samples obtained from different zones of a chronic wound. **a)** The graph shows the absorbance at 330 nm given by the content of amino acids of each sample (reference method).[¡Error! Marcador no definido.] **b)** Kinetic rate constants of displacement of the dye by amino acids of the samples *vs* absorbance at 330 nm obtained by the reference method.

S14. Reversibility of the material

The reversibility of the material was studied by the RGB method as explained below:

- <u>Charge & recharge</u>

The charging procedure was performed as depicted in Manuscript (Preparation of the sensory film).

- <u>Discharge</u>

Discs were dipped in a mixture of MeOH (1 mL), pH 7 buffer solution (1 mL) and 0.1 mL of Glycine 0,1 M for 1h.

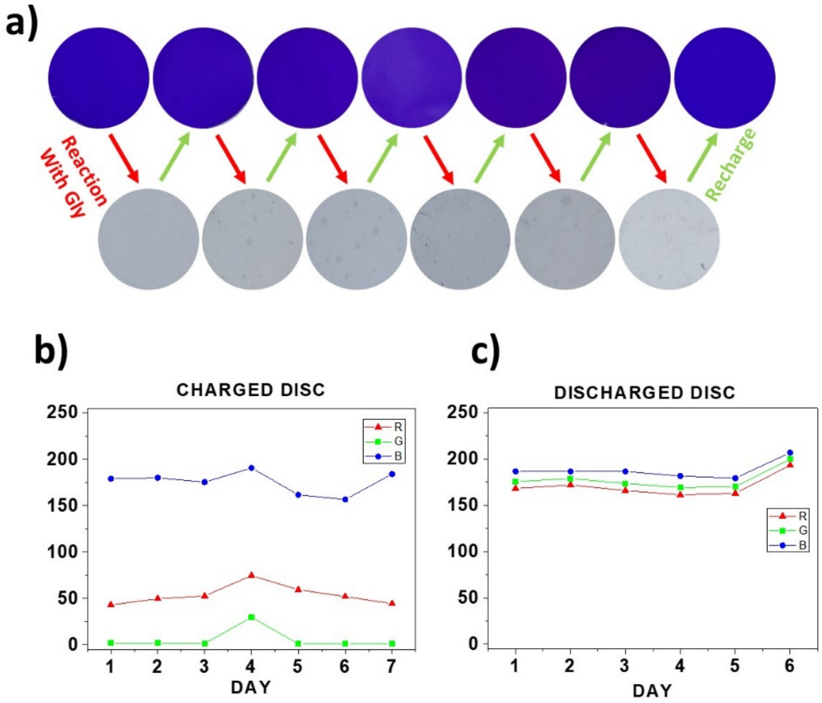

Figure S34. a) Photographs of $\mathbf{F_{(3)\text{-}Cu\text{-}D}}$ (blue) and $\mathbf{F_{(3)}}$ (colourless) discs after recharge and discharge process respectively. **b)** Colour evolution of RGB parameters of $\mathbf{F_{(3)\text{-}Cu\text{-}D}}$ discs after 7 charges/discharge cycles. **c)** Colour evolution of RGB parameters of $\mathbf{F_{(3)}}$ discs after 7 charges/discharge cycles.

References

1. S.K. Lagergren Zur Theorie der sogenannten Adsorption gelöster Stoffe - Lagergreen, S., (Bihang A. K. Svenske Vet. Ak. Handl. 24, II. Nr. 4, S. 49; 1899; Z. physik. Ch. 32, 174-75; 1900.). *Zeitschrift für Chemie und Industrie der Kolloide* **1907**, *2*, 15, doi:10.1007/BF01501332.
2. Ho, Y.S.; McKay, G. Pseudo-second order model for sorption processes. *Process Biochemistry* **1999**, *34*, 451–465, doi:10.1016/S0032-9592(98)00112-5.
3. Azizian, S. Kinetic models of sorption: A theoretical analysis. *Journal of Colloid and Interface Science* **2004**, *276*, 47–52, doi:10.1016/j.jcis.2004.03.048.
4. Crank, J. *Diffusion in a plane sheet*; Oxford University Press, 1975;
5. Chatterjee, A.; Schiewer, S. Multi-resistance kinetic models for biosorption of Cd by raw and immobilized citrus peels in batch and packed-bed columns. *Chemical Engineering Journal* **2014**, *244*, 105–116, doi:10.1016/j.cej.2013.12.017.
6. Boyd, G.E.; Adamson, A.W.; Myers, L.S. The Exchange Adsorption of Ions from Aqueous Solutions by Organic Zeolites. II. Kinetics. *Journal of the American Chemical Society* **1947**, *69*, 2836–2848, doi:10.1021/ja01203a066.
7. Reichenberg, D. Properties of Ion-Exchange Resins in Relation to their Structure. III. Kinetics of Exchange. *Journal of the American Chemical Society* **1953**, *75*, 589–597, doi:10.1021/ja01099a022.
8. Illanes, C.O.; Ochoa, N.A.; Marchese, J. Kinetic sorption of Cr(VI) into solvent impregnated porous microspheres. *Chemical Engineering Journal* **2008**, *136*, 92–98, doi:10.1016/j.cej.2007.03.008.
9. Choy, K.K.H.; McKay, G. Sorption of cadmium, copper, and zinc ions onto bone char using Crank diffusion model. *Chemosphere* **2005**, *60*, 1141–1150, doi:10.1016/j.chemosphere.2004.12.041.
10. Dávila-Guzman, N.E.; Cerino-Córdova, F.J.; Diaz-Flores, P.E.; Rangel-Mendez, J.R.; Sánchez-González, M.N.; Soto-Regalado, E. Equilibrium and kinetic studies of ferulic acid adsorption by Amberlite XAD-16. *Chemical Engineering Journal* **2012**, *183*, 112–116, doi:10.1016/j.cej.2011.12.037.
11. Sun, X.; Chen, J.H.; Su, Z.; Huang, Y.; Dong, X. Highly effective removal of Cu(II) by a novel 3-aminopropyltriethoxysilane functionalized polyethyleneimine/sodium alginate porous membrane adsorbent. *Chemical Engineering Journal* **2016**, *290*, 1–11, doi:10.1016/j.cej.2015.12.106.
12. Nielsen, P.M. Improved Method for Determining Protein Hydrolysis. *Journal of Food Science* **2001**, *66*, 642–646.
